# Extrauterine Placental Perfusion and Oxygenation in Infants With Very Low Birth Weight: A Randomized Clinical Trial

**DOI:** 10.1001/jamanetworkopen.2023.40597

**Published:** 2023-11-03

**Authors:** Benjamin Kuehne, Berthold Grüttner, Martin Hellmich, Barbara Hero, Angela Kribs, André Oberthuer

**Affiliations:** 1 Division of Neonatology, Department of Pediatrics, Faculty of Medicine and University Hospital Cologne, University of Cologne, Cologne, Germany; 2 Institute of Medical Statistics and Computational Biology, Faculty of Medicine and University Hospital Cologne, University of Cologne, Cologne, Germany; 3 Department of Gynecology and Obstetrics, Faculty of Medicine and University Hospital Cologne, University of Cologne, Cologne, Germany; 4 Department of Pediatrics, Faculty of Medicine and University Hospital Cologne, University of Cologne, Cologne, Germany

## Abstract

**Importance:**

An extrauterine placental perfusion (EPP) approach for physiological-based cord clamping (PBCC) may support infants with very low birth weight (VLBW) during transition without delaying measures of support.

**Objective:**

To test whether EPP in resuscitation of infants with VLBW results in higher hematocrit levels, better oxygenation, or improved infant outcomes compared with delayed cord clamping (DCC).

**Design, Setting, and Participants:**

This nonblinded, single-center randomized clinical trial was conducted at a tertiary care neonatal intensive care unit. Infants with a gestational age greater than 23 weeks and birth weight less than 1500 g born by cesarean delivery between May 2019 and June 2021 were included. Data were analyzed from October through December 2021.

**Intervention:**

Prior to cesarean delivery, participants were allocated to receive EPP or DCC. In the EPP group, infant and placenta, connected by an intact umbilical cord, were detached from the uterus and transferred to the resuscitation unit. Respiratory support was initiated while holding the placenta over the infant. The umbilical cord was clamped when infants showed regular spontaneous breathing, stable heart rates greater than 100 beats/min, and adequate oxygen saturations. In the DCC group, cords were clamped 30 to 60 seconds after birth before infants were transferred to the resuscitation unit, where respiratory support was started.

**Main Outcomes and Measure:**

The primary outcome was the mean hematocrit level in the first 24 hours after birth. Secondary prespecified outcome parameters comprised oxygenation during transition and short-term neonatal outcome.

**Results:**

Among 60 infants randomized and included, 1 infant was excluded after randomization; there were 29 infants in the EPP group (mean [SD] gestational age, 27 weeks 6 days [15.0 days]; 14 females [48.3%]) and 30 infants in the DCC group (mean [SD] gestational age, 28 weeks 1 day [17.1 days]; 17 females [56.7%]). The mean (SD) birth weight was 982.8 (276.6) g and 970.2 (323.0) g in the EPP and DCC group, respectively. Intention-to-treat analysis revealed no significant difference in mean hematocrit level (mean difference [MD], 2.1 percentage points; [95% CI, −2.2 to 6.4 percentage points]). During transition, infants in the EPP group had significantly higher peripheral oxygen saturation as measured by pulse oximetry (adjusted MD at 5 minutes, 15.3 percentage points [95% CI, 2.0 to 28.6 percentage points]) and regional cerebral oxygen saturation (adjusted MD at 5 minutes, 11.3 percentage points [95% CI, 2.0 to 20.6 percentage points]). Neonatal outcome parameters were similar in the 2 groups.

**Conclusions and Relevance:**

This study found that EPP resulted in similar hematocrit levels as DCC, with improved cerebral and peripheral oxygenation during transition. These findings suggest that EPP may be an alternative procedure for PBCC in infants with VLBW.

**Trial Registration:**

ClinicalTrials.gov Identifier: NCT03916159

## Key Points

**Question:**

Does a physiological-based cord-clamping approach by extrauterine placental perfusion (EPP) increase mean hematocrit levels or improve oxygenation or neonatal outcomes in resuscitation of infants with very low birth weight (VLBW) compared with time-based delayed cord clamping (DCC)?

**Findings:**

In this randomized clinical trial among 60 infants with VLBW, mean hematocrit levels did not differ between groups, but infants who received EPP had significantly improved peripheral and cerebral oxygenation during transition.

**Meaning:**

In this study, neonatal resuscitation of infants with VLBW using EPP resulted in comparable hematocrit levels as DCC but improved oxygenation during transition.

## Teaser

This randomized clinical trial investigates the effect of extrauterine placental perfusion compared with delayed cord clamping on mean hematocrit levels in infants with very low birth weight.

## Introduction

Transition from intrauterine to extrauterine life is a critical process in which clamping of the umbilical cord represents one of the most significant events, particularly for infants born preterm.^1,2^ The timing of clamping of the cord impacts the process of transition and infant outcomes. Delayed cord clamping (DCC) of at least 30 seconds compared with immediate cord clamping in infants born preterm has been shown to improve survival and reduce intraventricular hemorrhage (IVH) of any grade, rate of red blood cell transfusion, and risk of death or major disability at 2 years.^3,4,5^ However, the recommendation for DCC in infants born preterm is based mainly on studies that excluded infants who did not breathe spontaneously. Animal studies suggest that adequate aeration of the lungs before umbilical cord clamping, also termed physiological-based cord clamping (PBCC), may be the key for benefits of DCC.^6,7^ Although most infants with VLBW start to breathe within 30 to 60 seconds of DCC,^8^ initiation of spontaneous breathing is not equivalent to adequate aeration of the lungs and thus is not an indicator of successful transition.^9^ Over the last decade, mobile resuscitation units for PBCC have been developed to provide lung aeration in resuscitation of infants born preterm using continuous positive airway pressure (CPAP) during DCC. A study^10^ evaluating these devices revealed challenges in temperature management, space issues, and incomplete equipment sterility in the setting of cesarean delivery. Therefore, the applicability and effectiveness of such devices remain to be determined and alternative approaches for PBCC should be investigated.

It is assumed that by performing PBCC, blood from the placenta stabilizes hemodynamic transition from prenatal to postnatal circulation when the cord is not clamped prior to lung aeration. However, it is unknown if a placenta detached from the uterus has the same hemodynamic effects given that there is still ongoing placental circulation from the extrauterine placenta to the infant. Dunn^11^ studied the potential benefit of DCC after removal of the placenta from the mother in cesarean delivery of infants born preterm decades ago. This approach, in which infants are born by cesarean delivery with the placenta still connected via an intact umbilical cord and transferred to the resuscitation unit, may work as an alternative approach for PBCC. It was also shown that such an approach for PBCC is safe for mother and infant in babies born at term and provides placental perfusion independently of the mother.^12^ Our center has been using this approach in neonatal resuscitation of infants with VLBW born by cesarean delivery for more than a decade and has extended the method by simultaneously applying mask CPAP for respiratory support. We termed this modified approach extrauterine placental perfusion (EPP). A first retrospective analysis revealed that EPP was not associated with adverse outcomes for infants with VLBW.^13^

This is the first randomized clinical trial of EPP to our knowledge. We hypothesized that providing respiratory support with an intact umbilical cord by performing EPP for PBCC would increase mean hematocrit levels on the first day of life compared with DCC. In addition, we hypothesized that the EPP procedure would stabilize the circulation during neonatal transition and would not adversely affect short-term neonatal outcomes.

## Methods

### Study Site and Recruitment

The Extrauterine Placental Transfusion in Resuscitation of VLBW Infants (EXPLAIN) trial (German Clinical Trial register: DRKS00017041; ClinicalTrials.gov: NCT03916159) was a nonblinded, single-center randomized clinical trial conducted at the University Hospital of Cologne, Germany (see trial protocol and statistical analysis plan in Supplement 1). The EXPLAIN study was approved by the local ethics committee of the medical faculty of the University of Cologne. Parents of infants included in the EXPLAIN trial provided informed consent. Adverse outcomes and adverse events of special interest were reviewed by the steering committee 4 times per year. Data quality was assured by external monitoring by the Clinical Trials Center Cologne. This article follows the current Consolidated Standards of Reporting Trials (CONSORT) reporting guideline.

Pregnant individuals with a gestational age more than 23 6/7 weeks and an estimated fetal weight of less than 1500 g who were at risk of preterm delivery were eligible for this study. Parents were approached for informed consent prior to delivery. Only infants born by cesarean delivery were eligible for this study. Exclusion criteria included vaginal delivery, fetal or maternal risk (ie, compromise, emergency cesarean delivery, or general anesthesia), placental abruption or placenta previa with hemorrhage, placenta accreta or increta, monochorionic multiples, congenital anomalies, and lack of consent to the study from parents.

### Randomization

Participants were randomly assigned using opaque, sealed envelopes, stratified by gestational age (24 0/7 to 27 6/7 weeks or ≥28 0/7 weeks) and type of pregnancy (singleton or twin pregnancy). The allocation sequence was based on computer-generated pseudorandom numbers (permuted blocks of varying length). The research team opened envelopes in the theater immediately before the cesarean delivery. The randomization result was then announced to the entire team during the team time-out prior to the start of the surgery.

### Procedures

Infants randomized to the control group (DCC) were born by cesarean delivery, placed in a sterile plastic suit (NeoHelp, Vygon), and gently stimulated by rubbing the back or feet if apneic until breathing commenced. Infants were held at placental level during the DCC procedure (eFigure in Supplement 2). Onset of breathing or first crying and time from delivery until the umbilical cord was clamped were recorded. Minimum time until clamping the cord was 30 seconds. Immediately after cord clamping, mothers received oxytocin, followed by manual detachment of the placenta. Once the cord was clamped, infants were transferred to the resuscitation unit, where neonatal staff (including B.K., A.K., and A.O.) supported the infant’s transition according to our unit’s protocol, as described previously.^14,15^ Briefly, all infants received mask CPAP starting with a positive airway pressure application of 8 to 10 cm H_2_O with stepwise increase up to a maximum of 30 cm H_2_O applied using the Benveniste valve as CPAP generator. Initially, fraction of inspired oxygen (Fio_2_) was set in the range of 0.21 to 0.30 in infants at less than 28 weeks’ gestational age.^14,16^

Infants randomized to the intervention group (EPP) were born by cesarean delivery with gentle detaching of the placenta from the uterus along with the infant and intact umbilical cord. All involved obstetricians and surgical staff (including B.G.) were experienced in this technique. After delivery, infants with the placenta still attached were placed in a sterile plastic suit by the obstetrician. The time to detach the placenta in the EPP cohort was not recorded but comprised only a few seconds. While mothers received oxytocin directly after delivery of the placenta, infants were immediately transferred to the resuscitation unit, where the neonatal staff (including B.K., A.K., and A.O.) resuscitated the infant according to the same protocol as described in control group. The placenta was placed in a bowl, covered with a sterile plastic bag, and held 40 to 50 cm above the babies’ heart level while lung recruitment by respiratory support was performed (eFigure in Supplement 2). The umbilical cord was clamped and cut when infants presented a regular respiratory pattern, a stable heart rate of greater than 100 beats/min, and an increasing pulse oxygen saturation (according to the reference range by Dawson et al^17^).

Placental weight was measured immediately after initiation of neonatal resuscitation and after umbilical cord clamping. Furthermore, onset of breathing or first crying and time from delivery until clamping of the umbilical cord were recorded in the intervention group.

Oxygen saturation as measured by pulse oximetry (Spo_2_) and heart rate were measured with a Masimo 7 SET pulse-oximeter (Masimo Radical, Masimo Corporation). Cerebral oxygenation was measured by near-infrared spectroscopy (Foresight, Casmed), with sensors placed on infants’ foreheads after transfer to a resuscitation bed. Regional cerebral oxygen saturation (rcSo_2_) was recorded synchronously with cardiorespiratory parameters.

When possible, a New Life Box ALD resuscitation monitor (Advanced Life Diagnostics) additionally recorded respiratory rate, airway pressure, and tidal volume in both groups during neonatal resuscitation. In all twin births, only the second newborn was randomized to the control or intervention group, while the firstborn was always intended to have a DCC of at least 30 seconds.

### Primary and Secondary Outcomes

In our center, hematocrit level is routinely determined in every infant with VLBW at 15 to 60 minutes after birth and at 12 and 24 hours after birth. The primary outcome was the mean value of these measurements during the first 24 hours after birth.

In addition, prespecified secondary outcomes, including heart rate, Spo_2_ and rcSo_2_ levels during transition, neonatal and maternal complications, and parameters related to the study procedure, were collected from infant medical records. All study data were collected and managed using REDCap electronic data-capture tools.^18,19^ Bronchopulmonary dysplasia was categorized according to the definition of Walsh et al.^20^ A summary of secondary outcome parameters is provided in the study protocol (Supplement 1). Results of a priori–defined secondary outcome parameters with respect to cytokine profiles in blood samples and neurodevelopmental assessment at age 2 years will be reported separately.

### Sample Size Calculation

We hypothesized that EPP would increase the mean (SD) hematocrit level in the first 24 hours after birth by a relative 10%, from 52.1% (6.6%) in the DCC group (see Katheria et al^21^) to 57.3% (7.3%) in the EPP group. The 2-sample *t* test requires a sample size of 2 × 29 evaluable patients (ie, approximately 60 patients) to reach a power of 80% at a 2-sided significance level of 5%. We used the power twomeans command in Stata statistical software version 15.1 (StataCorp).

### Statistical Analysis

The primary analysis was by intention to treat, including all randomized patients who were evaluated according to their assigned intervention. The primary outcome was the mean hematocrit level in the first 24 hours after birth. Group comparison (based on adjusted mean difference [AMD]) was performed by analysis of covariance (ANCOVA) with fixed effects for group, gestational age (24 weeks 0 days to 27 weeks 6 days vs >27 weeks 6 days) and gravidity (singleton vs multiples). Quantitative variables were described with the number of valid and missing values, mean, SD, and percentiles (0%, 25%, 50%, 75%, and 100%); group comparison was done analogously to the primary outcome. Qualitative variables are reported with absolute (number) and relative (percentage) frequencies; group comparison was done based on relative risk (with 95% CIs). A 2-tailed *P* value ≤ .05 was considered statistically significant. Data were analyzed from October through December 2021.

## Results

**Figure 1.**
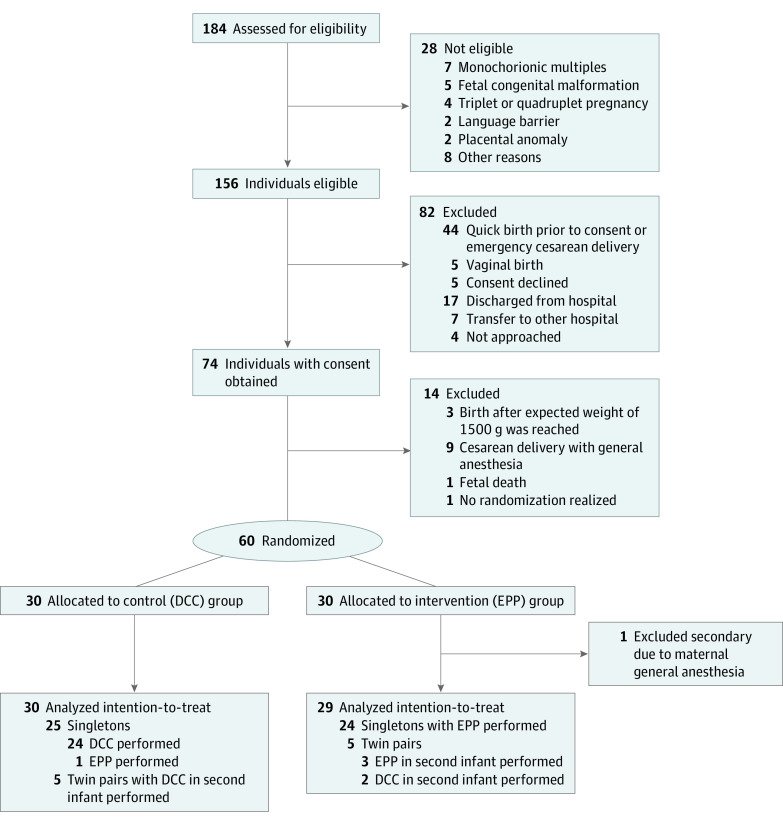
Study Flowchart DCC indicates delayed cord clamping; EPP, extrauterine placental perfusion.

Among a total of 156 eligible pregnant individuals, 60 individuals were randomized and enrolled in this study between May 2019 and June 2021. A total of 30 individuals were assigned to the control group (DCC) and 30 to the intervention group (EPP), respectively (Figure 1). One newborn assigned to the intervention group was excluded after randomization because the mother was under general anesthesia, resulting in a study population of 59 infants (mean [SD] gestational age, 28 weeks 0 days [2.1 days]; 31 females [52.5%]), including 29 infants in the EPP group (mean [SD] gestational age, 27 weeks 6 days [15.0 days]; 14 females [48.3%]) and 30 infants in the DCC group (mean [SD] gestational age, 28 weeks 1 day [17.1 days]; 17 females [56.7%]). The mean (SD) birth weight was 982.8 (276.6) g and 970.2 (323.0) g in the EPP and DCC group, respectively. Baseline clinical characteristics were similar in the 2 groups (Table 1).

**Table 1.** Participant Characteristics

Characteristic	Participants, No. (%) (N = 59)^a^	
	EPP (n = 29)	DCC (n = 30)
**Infants**		
Gestational age, mean (SD), wk + d (d)	27 + 6 (15.0)	28 + 1 (17.1)
Birth weight, mean (SD), g	982.8 (276.6)	970.2 (323.0)
Sex		
Female	14 (48.3)	17 (56.7)
Male	15 (51.7)	13 (43.3)
Multiple birth	5 (17.2)	5 (16.7)
**Mothers**		
Age, median (IQR), y	33 (29-37)	34.5 (29-36)
Antenatal corticosteroids	28 (96.6)	26 (86.7)
Partial course	3 (10.7)	7 (26.9)
Full course	25 (89.3)	19 (73.1)
PROM		
Total with data, No.	25	28
Overall	8 (32.0)	8 (28.6)
1-23 h Before delivery	0	4 (14.3)
>24 h Before delivery	8 (32.0)	4 (14.3)
Preeclampsia	2 (6.9)	9 (30.0)
HELLP	1 (3.4)	3 (10.0)
Active labor	11 (37.9)	8 (26.7)

Abbreviations: DCC, delayed cord clamping; EPP, extrauterine placental perfusion; HELLP, hemolysis, elevated liver enzymes, and low platelet count; PROM, prolonged rupture of membranes.

^a^ Intention-to-treat set.

In the intervention group EPP was feasible in 24 of 24 singletons and in 3 of 5 second twins. In 1 twin pair, EPP was not feasible because the second twin’s placenta was attached to first twin’s placenta; the procedure was switched to DCC according to the study protocol. In the other case of twin birth, the attending obstetrician opted for immediate cord clamping in both infants due to severe chorioamnionitis and maternal instability. In the control group, DCC was performed as randomized in 24 of 25 singletons and 5 of 5 twin births. In 1 case, the placenta was accidentally detached during cesarean delivery before birth, and thus the infant received EPP instead of DCC.

**Figure 2.**
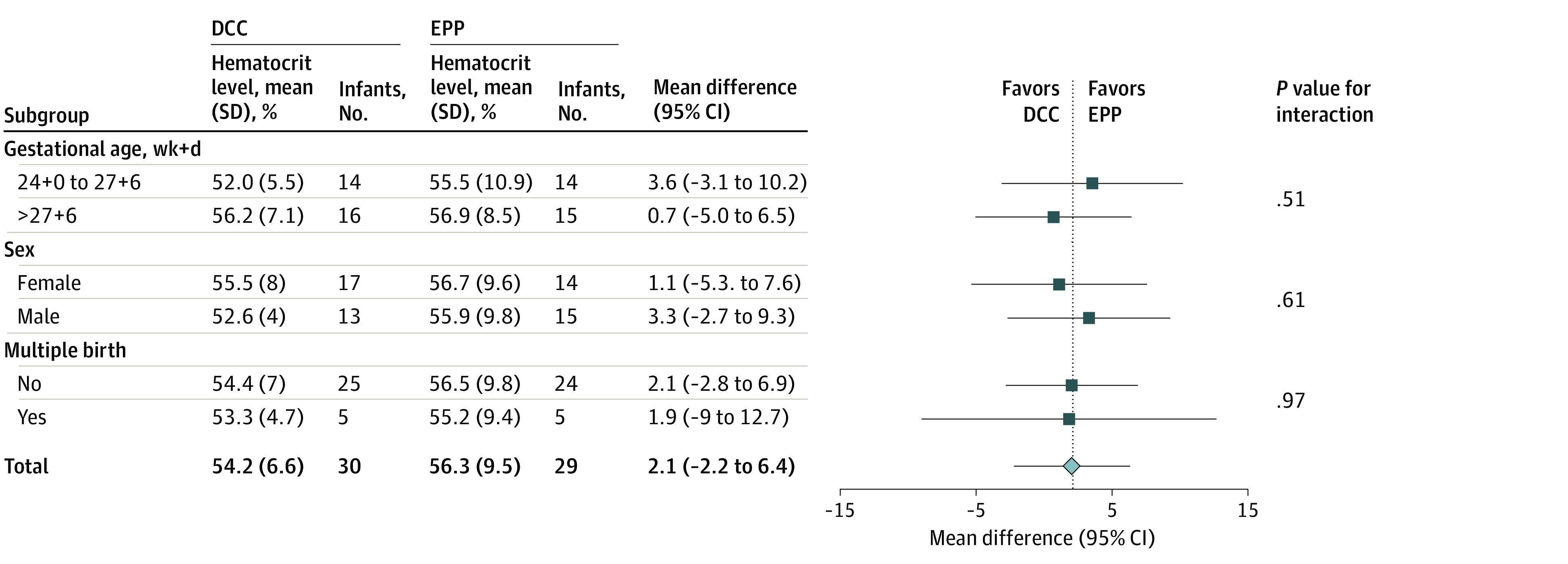
Subgroup Analyses for Gestational Age, Sex, and Mode of Pregnancy DCC indicates delayed cord clamping; EPP, extrauterine placental perfusion.

**Figure 3.**
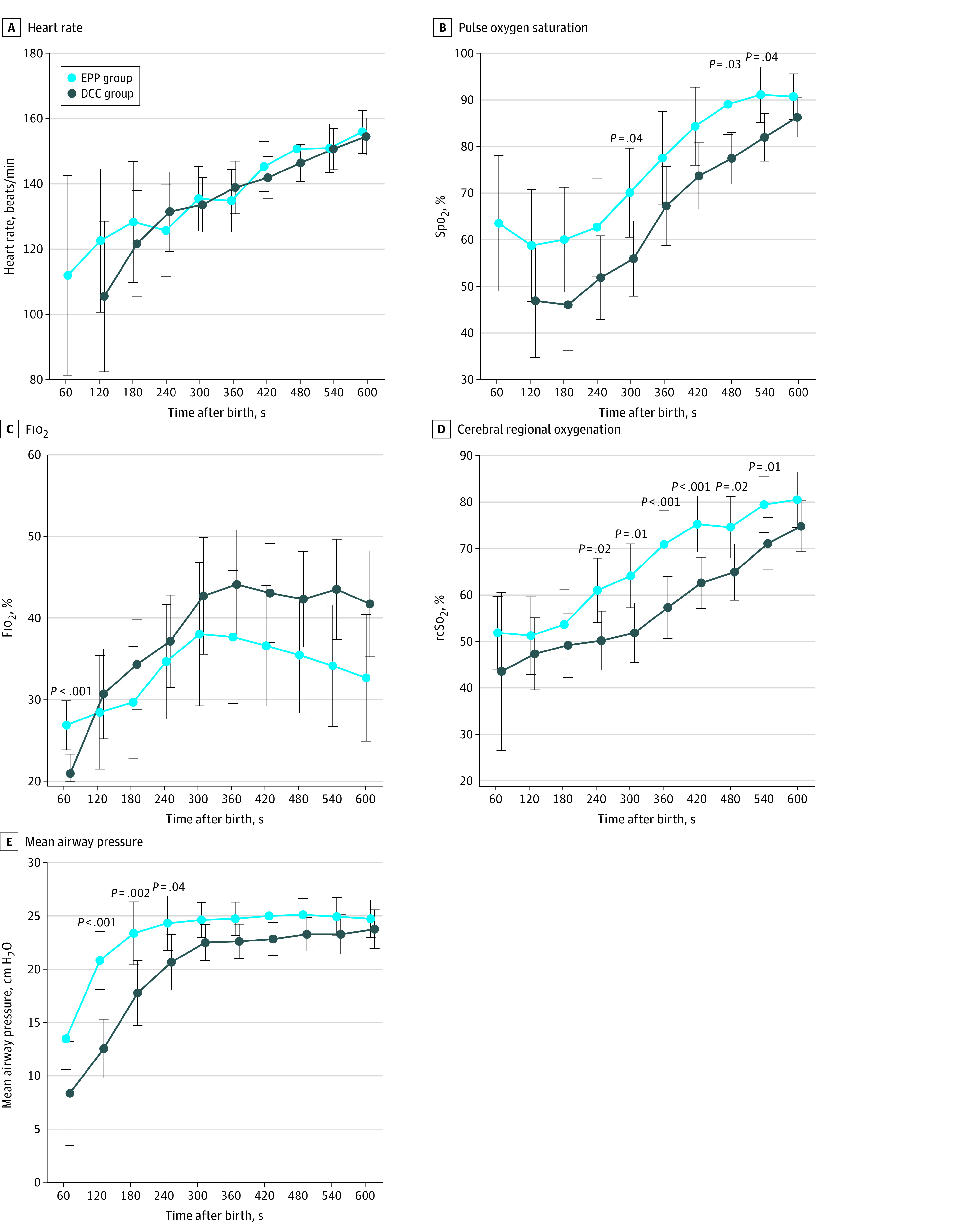
Secondary Outcomes During Neonatal Transition Values were picked at the precise time indicated from data of continuous monitoring and are presented as means and 95% CIs (whiskers). A mixed model for repeated measures with fixed effects for group, time, interaction group × time, and a first-order autoregressive–structured covariance matrix over time was fitted. Comparisons between groups at specific times were based on pairwise contrasts of marginal means, with 95% CIs and *P* values. DCC indicates delayed cord clamping; EPP, extrauterine placental perfusion; Fio_2_, fraction of inspired oxygen; Spo_2_, oxygen saturation as measured by pulse oximetry; rcSo_2_, regional cerebral oxygen saturation.

The intention-to-treat analysis revealed no significant difference in the primary outcome. In the EPP group, the mean (SD) hematocrit level was 56.0% (1.8%) compared with 53.9% (1.8%) in the DCC group (MD, 2.1 percentage points [95% CI, −2.2 to 6.4 percentage points]). Subgroup analyses by gestational age, sex, and mode of pregnancy also showed no significant differences (Figure 2). With respect to the prespecified secondary outcome criteria, infants in the intervention group had significantly higher mean (SD) Spo_2_ levels (age 5 minutes: 70.1% [26.1%] vs 55.7% [17.9%]; AMD, 15.3 percentage points [95% CI, 2.0 to 28.6 percentage points]; age 8 minutes: 88.9% [10.4%] vs 78.3% [17.8%]; AMD, 11.3 percentage points [95% CI, 2.0 to 20.6 percentage points]) (Figure 3B; eTable 1 in Supplement 2) and rcSo_2_ levels (age 5 minutes: 65.3% [15.6%] vs 51.9% [17.1%]; AMD, 13.2 percentage points [95% CI, 2.4 to 24.0 percentage points]); age 8 minutes: 76.3% [14.5%] vs 65.1 [15.9%]; AMD, 10.9 percentage points [95% CI, 1.5 to 20.3 percentage points]) (Figure 3D; eTable 2 in Supplement 2) during the transition, although Fio_2_ levels were lower in the EPP group (Figure 3C; eTable 3 in Supplement 2) during neonatal transition. Applied mean airway pressure at the onset of neonatal transition was statistically significantly higher in the EPP group compared with the DCC group (Figure 3E). There were no significant differences in other predefined neonatal outcome parameters (Table 2). A significantly higher mean (SD) maternal blood loss during cesarean delivery was observed in the intervention group vs the control group (488.9 [229.7] mL vs 409.6 [247.0]; *P* = .05) but was not confirmed by ANCOVA (AMD, 80.4 mL [95% CI, −42.7 to 203.5 mL]) (Table 2). A secondary per-protocol analysis was performed and yielded similar results (eTables 4-7 in Supplement 2).

**Table 2.** Neonatal and Maternal Outcomes^a^

Outcome	Participants, No. (%) (N = 59)		Outcome (95% CI)^b^
	EPP (n = 29)	DCC (n = 30)	
Apgar score, median (IQR)			
5 min	7 (7 to 8)	8 (7 to 8)	AMD: −0.2 (−0.7 to 0.3)
10 min	8 (8 to 9)	9 (8 to 9)	AMD: −0.3 (−0.6 to 0.1)
Time to onset of breathing			
Total with data, No.	25	20	NA
Mean (SD), s	9.8 (9.9)	14.1 (23.7)	AMD: −4.7 (−13.9 to 4.5)
Time of umbilical cord clamping, mean (SD), s	484.2 (169.8)	39.0 (8.2)	AMD: 445.8 (381 to 510.5)
Weight difference in placenta before vs after EPP			
Total with data, No.	23	NA	NA
Mean (SD), g	19.9 (9.7)	NA	NA
Respiratory support in delivery room			
CPAP	29 (100)	30 (100)	NA
NIPPV	0	0	NA
Intubation and mechanical ventilation	0	0	NA
Surfactant given in delivery room	28 (96.6)	27 (90.0)	RR: 0.4 (0.0 to 3.1)
Admission temperature, mean (SD), °C	36.5 (0.7)	36.3 (0.8)	AMD: 0.2 (−0.2 to 0.6)
Blood transfusion during first 7 d of life	1 (3.4)	1 (3.3)	RR: 1.0 (0.1 to 1.1)
Peak levels			
IL-6 in first 24 h, median (IQR), ng/L	31.5 (10.5 to 49.0)	8.5 (4.0 to 25.0)	AMD: 240.2 (−138.5 to 619.0)
CRP in first 24 h, median (IQR), mg/dL	0.16 (0.1 to 0.2)	0.11 (0.1 to 2.0)	AMD: 1.4 (−1.3 to 4.1)
Bilirubin in first 14 d, mean (SD), mg/dL	9.9 (2.3)	9.8 (2.5)	AMD: 0.1 (−0.9 to 1.2)
Duration of phototherapy, mean (SD), d	4.8 (2.7)	4.8 (2.3)	AMD: −0.1 (−1.5 to 1.2)
Exchange transfusion due to severe hyperbilirubinemia	0	0	NA
Intubation			
In first 72 h	3 (10.3)	3 (10.0)	RR: 1.0 (0.8 to 1.2)
Until discharge	6 (20.7)	5 (16.7)	RR: 1.0 (0.8 to 1.2)
Duration of mechanical ventilation, median (IQR), h	318.5 (219.0 to 912.0)	238.0 (167.0 to 267.0)	AMD: 272.1 (−352.3 to 896.5)
Pneumothorax			
In first 7 d of life	1 (3.4)	2 (6.7)	RR: 1.0 (0.9 to 1.2)
Until discharge	1 (3.4)	2 (6.7)	RR: 1.0 (0.9 to 1.2)
Spontaneous intestinal perforation			
Without surgery	0	3 (10.0)	RR: 1.1 (1.0 to 1.3)
With surgery	2 (6.9)	3 (10.0)	RR: 1.0 (0.9 to 1.2)
Necrotizing enterocolitis			
Without surgery	0	0	NA
With surgery	2 (6.9)	0	RR: 0.9 (0.8 to 1.0)
Intraventricular hemorrhage			
Grade 1	4 (13.8)	6 (20.0)	RR: 1.1 (0.8 to 1.4)
Grade 2	1 (3.4)	0	RR: 1.0 (0.9 to 1.0)
Grade ≥3	2 (6.9)	0	RR: 0.9 (0.8 to 1.0)
Periventricular leukomalacia			
Total with data, No.	28	30	NA
Yes	1 (3.6)	0	RR: 1.0 (0.9 to 1.0)
Bronchopulmonary dysplasia at 36 wk corrected			
Total with data, No.	26	30	NA
Mild	18 (69.2)	15 (51.7)	RR: 0.6 (0.3 to 1.3)
Moderate	0	2 (6.7)	RR: 1.2 (1.0 to 1.4)
Severe	1(1.8)	0	RR: 0.9 (0.7 to 1.1)
Retinopathy necessitating intervention (medical or surgical)			
Total with data, No.	28	30	NA
Yes	1 (3.6)	1 (3.3)	RR: 1.0 (0.9 to 1.1)
Survival until discharge	28 (96.6)	30 (100)	RR: 1.0 (0.9 to 1.0)
Lowest maternal Hb in first 24 h after delivery, mean (SD), g/dL	10.2 (1.0)	10.4 (1.6)	AMD: −0.2 (−0.9 to 0.5)
Maternal blood loss during cesarean surgery			
Total with data, No.	27	26	NA
Mean (SD), mL	488.9 (229.7)	409.6 (247)	AMD: 80.4 (−42.7 to 203.5)
Maternal postpartum hemorrhage >1000 mL			
Total with data, No.	27	26	NA
Yes	1 (3.7)	1 (3.8)	RR: 1.0 (0.9 to 1.1)
Chorioamnionitis with maternal CRP >2.0 mg/dL	4 (13.8)	1 (3.3)	RR: 0.9 (0.8 to 1.1)
Maternal survival until discharge	29 (100)	30 (100)	NA

Abbreviations: AMD, adjusted mean difference; Apgar, appearance, pulse, grimace, activity, and respiration; CPAP, continuous positive airway pressure; CRP, C-reactive protein; DCC, delayed cord clamping; EPP, extrauterine placental perfusion; Hb, hemoglobin; IL, interleukin; NA, not applicable; NIPPV, nasal intermittent positive pressure ventilation; RR, relative risk.

SI conversion factors: To convert bilirubin to micromoles per liter, multiply by 17.104; CRP to milligrams per liter or Hb to grams per liter, multiply by 10.

^a^ Intention-to-treat set.

^b^ AMDs (with 95% CIs) were estimated by univariate analysis of variance, with cofactors (gestational age: 24 weeks 0 days to 27 weeks 6 days and >27 weeks 6 days and type of pregnancy: singleton and multiple).

## Discussion

To our knowledge, this is the first randomized clinical trial of EPP as an alternative approach for PBCC instead of DCC in infants with VLBW. Our study found that the EPP approach resulted in similar mean hematocrit levels 24 hours after birth and was safe given similar neonatal outcomes in EPP and DCC groups. While numerous studies have shown a significantly higher hematocrit level after DCC in babies born spontaneously, studies in infants born by cesarean delivery are controversial.^22,23^ The absence of uterine contractions during elective cesarean delivery and the cutting of the uterus during this type of delivery itself may contribute to a generally reduced placental transfusion in infants delivered by this method.^24^ Of note, most infants in both groups studied in our trial were delivered by elective cesarean delivery. However, it was interesting to us that infants receiving EPP, in whom the cord was clamped after successful lung aeration, did not show higher hematocrit levels in the first 24 hours after birth compared with infants receiving DCC. While lung aeration prior to cord clamping is thought to be responsible for a significantly greater amount of transfused blood,^6^ most studies have failed to find higher postnatal hematocrit levels in infants after PBCC, which is consistent with our findings.^21,25^

An intriguing observation of our study was that infants receiving EPP had significantly higher Spo_2_ and rcSo_2_ levels during transition than infants receiving DCC. A faster increase in arterial oxygenation in the setting of PBCC has been observed in animal studies^26^ and confirmed in a clinical study of PBCC in infants born preterm.^27^ In addition to these observations, we found significantly higher cerebral oxygenation during the first minutes after birth. Prolonged placental perfusion in PBCC optimizes tissue perfusion and may influence cerebral perfusion, with the potential to reduce the risk of IVH.^28^ Our study demonstrates that this effect is also observed in PBCC with an extrauterine placental circulation. This supports the hypothesis that the main benefit of PBCC with sustained placental perfusion may not be in the higher number of red blood cells but in the provision of preload volume stabilizing cardiac output during neonatal transition. This may be the key to avoiding disturbances in cerebral blood flow and preventing neonatal complications. In a 2020 study, Padilla-Sánchez et al^29^ found significantly higher Spo_2_ values in infants who were mature and late preterm with sufficient respiration receiving DCC compared with reference values in infants who underwent immediate cord clamping. This underscores the importance of successful lung aeration and adequate placental perfusion during transition. Our finding that infants in the EPP group showed better oxygenation parameters raises another interesting question: whether oxygen transfer from mother to infant across the placenta, which does not occur in EPP because the placenta is detached, may be less important than an effective maintenance of venous return to the heart, which is supported by our EPP approach.

In this trial, the EPP approach was feasible in all singleton and most multiple births. This finding is consistent with published data in neonates born at term^12^ and supports the finding that EPP may be considered as an alternative to the currently recommended practice of DCC.

Compared with PBCC approaches using mobile beside resuscitation platforms, EPP has several advantages: first, the procedure does not delay the course of cesarean delivery compared with DCC, in which surgical procedures are stopped until the cord is clamped. Although we did not record time from delivery of the infant to placental detachment, data from Welsh et al^12^ and our own experience with the EPP procedure showed that times were much shorter than in DCC when staff were experienced with the technique. Second, the procedure does not conflict with the sterility of the operating room.^30^ Third, most current resuscitation platforms are not certified for transport, requiring transfer of the infant after initial stabilization.^10^ Fourth, the EPP approach is a cost-effective and technically simple approach for PBCC. Therefore, it may be particularly attractive in resource-limited settings where mobile resuscitation units are not available. Moreover, a 2021 survey^31^ confirmed the need for alternative procedures to achieve placental perfusion given that delayed cord clamping was performed in 60% of infants born preterm who were stable and 6% of infants born preterm who were unstable in low- and middle-income countries.

Although there was a statistically significant difference in maternal blood loss during cesarean delivery, the higher blood loss in mothers in the EPP compared with DCC group is not considered clinically significant by obstetricians. In addition, significance was not confirmed in ANCOVA. The higher rate of blood loss in mothers in the EPP group may be explained by differences in the surgical procedure when performing EPP. Whereas in EPP oxytocin is administered after the placenta has been detached, in DCC it is usually administered immediately after umbilical cord clamping but before the placenta has been detached. Further studies on the EPP technique should address the issue of maternal blood loss in terms of safety.

### Limitations

Our EXPLAIN study has several limitations. First, restricting a study to participants giving prenatal consent may limit external validity because mothers who were not approached were in advanced labor. Thus, a large proportion of infants at a higher risk of neonatal complications may not have been enrolled, also resulting in a relatively old patient population. Second, this was a monocentric trial comprising 60 infants in a center with long experience in EPP and a different strategy of respiratory support during neonatal resuscitation based on titration of high continuous airway pressures rather than the use of positive pressure ventilation. Therefore, our findings regarding feasibility may not be generalizable to other settings.

Another important limitation of our trial is that we did not calculate sample size with regard to safety aspects of the EPP procedure. Therefore, our study did not have sufficient power to conclude with certainty that these 2 interventions were interchangeable with respect to adverse maternal and neonatal outcomes in the short and long term. Given that the incidence of major neonatal complications, such as bronchopulmonary dysplasia, IVH, or necrotizing enterocolitis, is low in our center, a higher number of participants may be needed to detect significant differences in these outcome variables. In this context, further multicenter trials are mandatory to determine feasibility and reproducibility in other centers less experienced with EPP.

## Conclusions

Findings from this first monocentric randomized clinical trial of EPP suggest that it may be an alternative procedure for PBCC in infants with VLBW. EPP resulted in similar postnatal hematocrit levels compared with DCC but significantly higher peripheral and cerebral oxygenation levels during transition. Further, larger trials are needed to evaluate the impact of EPP on neonatal outcomes.

## Notes

Supplement 1.
Trial Protocol and Statistical Analysis Plan

Supplement 2. **eFigure.** Umbilical Cord Management for Infants of the Control and Intervention Group **eTable 1.** Heart Rate and Pulse Oxygen Saturation During Neonatal Transition (Intention-to-Treat Analysis) **eTable 2.** Cerebral Regional Oxygenation During Neonatal Transition (Intention-to-Treat Analysis) **eTable 3.** Airway Pressure and Fraction Of Inspired Oxygen Levels During Neonatal Transition (Intention-to-Treat Analysis) **eTable 4.** Neonatal and Maternal Outcomes (Per-Protocol Analysis) **eTable 5.** Heart Rate and Pulse Oxygen Saturation During Neonatal Transition (Per-Protocol Analysis) **eTable 6.** Cerebral Regional Oxygenation During Neonatal Transition (Per-Protocol Analysis) **eTable 7.** Airway Pressure and Fraction Of Inspired Oxygen Levels During Neonatal Transition (Per-Protocol Analysis)

Supplement 3.
Data Sharing Statement
