# Supplementary material for: Extrauterine Placental Perfusion and Oxygenation in Infants With Very Low Birth Weight: A Randomized Clinical Trial
Source: JAMA Netw Open. 2023 Nov 3;6(11):e2340597. doi: 10.1001/jamanetworkopen.2023.40597 (PMC10625045; doi:10.1001/jamanetworkopen.2023.40597)
Supplement: Supplement 1. — Trial Protocol and Statistical Analysis Plan [file jamanetwopen-e2340597-s001.pdf]

Clinical Trial Protocol

**Study Title:**	**Extrauterine Placental transfusion In Neonatal resuscitation (EXPLAIN) of very low birth weight infants (VLBW): A randomized clinical trial**
**Protocol Number:**	**EXPLAIN**
**Protocol Version:**	**1.4**
**Protocol Date:**	**17 May 2020**
**Clinicaltrials.gov:**	**Register Number NCT03916159**
**German Clinical Trials:**	**Register Number DRKS00017041**
**Sponsor:**	**University hospital of Cologne, Kerpener Str. 62, 50937 Cologne, Germany**

This document is a confidential communication of University hospital of Cologne. Acceptance of this document constitutes agreement by the recipient that no unpublished information contained herein will be published or disclosed without prior written approval, with the exception of disclosure to the appropriate Institutional Review Board/Independent Ethics Committee and/or Regulatory Authority under the condition that confidentiality is maintained

1 TABLE OF CONTENTS

2 ABBRIVIATIONS 4
3 SIGNATURES 5
4 SYNOPSIS 6
5 RESPONSIBILITIES 8
5.1 KEY TRIAL CONTACTS 8
5.2 CO-INVESTIGATORS 8
5.3 STEERING COMMITTEE 8
5.4 PARTICIPATING INSTITUTIONS / STUDY CENTER / INVESTIGATORS / EXTERNAL STUDY MONITORING 9
5.5 TRIAL REGISTER 10
6 BACKGROUND 11
6.1 CLINICAL PART 11
6.2 EXPERIMENTAL PART 12
6.3 RATIONAL AND JUSTIFICATION FOR THE STUDY 12
7 PROJECT OBJECTIVES 14
7.1 HYPOTHESIS AND PRIMARY OBJECTIVE 14
7.2 HYPOTHESIS AND SECONDARY OBJECTIVES 14
7.3 FEASIBILITY 14
8 STUDY OBJECTIVES 15
8.1 PRIMARY OUTCOME 15
8.2 SECONDARY OUTCOME 15
8.3 STUDY DESIGN 15
9 STUDY POPULATION 15
9.1 INCLUSION CRITERIA 16
9.2 EXCLUSION CRITERIA 16
9.3 NUMBER OF PARTICIPANTS 16
9.4 METHODS FOR ASSIGNING PARTICIPANTS TO TREATMENT GROUPS 16
9.5 SUBSEQUENT EXCLUSION OF STUDY PARTICIPANTS 16
9.6 PROCEDURE IN CASE OF NON-FEASIBILITY OF THE INTERVENTION 17
9.7 APPROACH TO DICHORIAL TWIN PREGNANCY 17
9.8 PROCEDURE IN CASE OF MISSING HEMATOCRIT MEASUREMENT 17
10 STUDY PROCEDURE 18
10.1 SCREENING AND ELIGIBILITY ASSESSMENT 18
10.2 PROCEDURE IN CONTROL/INTERVENTION GROUP 19
11 OVERALL DURATION OF THE TRIAL 26
12 BENEFIT RISK CONSIDERATION 27
12.1 INDIVIDUAL BENEFITS ASSOCIATED WITH STUDY PARTICIPATION 27

12.2 RISKS AND EXPOSURES ASSOCIATED WITH STUDY PARTICIPATION 27
12.3 RECORDING TYPICAL COMPLICATIONS OF PREMATURITY 27
_12.4_ CRITERIA FOR PREMATURE TERMINATION OF THE STUDY 28
13 MODIFICATIONS IN THE STUDY PROTOCOL 28
14 STATISTIC 29
14.1 STATISTICAL AND ANALYTICAL PLAN 29
14.2 STUDY POPULATION 29
14.3 PRIMARY TARGET VARIABLE 29
14.4 SECONDARY TARGET VARIABLES 29
14.5 EXPERIMENTAL PART 32
14.6 SUBGROUP ANALYSIS 33
14.7 SAMPLE CALCULATION 33
15 DATA MANAGEMENT 34
15.1 DATA RECORDING 34
15.2 DATA MANAGMENT 34
_15.3_ DATA PROTECTION 34
16 BIOMATERIALS MANAGEMENT 35
17 INSURANCE 36
18 PUBLICATION POLICY 37
19 REFERENCES 38

2 ABBRIVIATIONS

BPD	-	bronchopulmonary dysplasia
BSID	-	bayley scales of infant development
CM	-	cord milking
CMV	-	cytomegalie virus
CPAP	-	continuous-positive-airway-pressure
CTG	-	cardiotocography
DCC	-	delayed cord clamping
EK	-	red blood cell concentrate
EPT	-	extrauterine placental transfusion
FiO2	-	fraction of inspired oxygen
SIP	-	spontaneous intestinal perforation
Hb	-	hemoglobin
HELLP	-	hemolysis - elevated liver enzyme levels- low platelet count
Hkt	-	hematocrit
ICC	-	immediate cord clamping
IVH	-	intraventricular hemorrhage
NEC	-	necrotizing enterocolitis
NIRS	-	near-infrared spectroscopy
PCR	-	polymerase chain reaction
PEEP	-	positive end expiratory pressure
PVL	-	periventricular leukomalacia
$rScO_2$	-	regional cerebral oxygen saturation
ROP	-	retinopathia praematurorum
VLBW	-	very low birth weight

3 SIGNATURES

Priv.-Doz. Dr. med. André Oberthür	______________________	__________
Principal investigator	Signature	Date
Priv.-Doz. Dr. med. Angela Kribs	______________________	__________
Co-Principal investigator	Signature	Date
Dr. med. Benjamin Kühne	______________________	__________
Project management	Signature	Date
Prof. Dr. Martin Hellmich	______________________	__________
Statistics	Signature	Date

4 SYNOPSIS

**TITLE**	**EX**trauterine **PLA**cental transfusion **I**n **N**eonatal resuscitation (**EXPLAIN**) – a randomized clinical trial
**RESPONSIBILITY**	Priv.-Doz. Dr. med. André Oberthür (Principal Investigator)<br>University hospital of Cologne, Department of Pediatric and Adolescent Medicine, Neonatology and Pediatric Intensive Care Medicine, Kerpener Str. 62, 50937 Cologne, Germany<br><br>Priv.-Doz. Dr. med. Angela Kribs (Co-Principal Investigator)<br>University hospital of Cologne, Department of Pediatric and Adolescent Medicine, Neonatology and Pediatric Intensive Care Medicine, Kerpener Str. 62, 50937 Cologne, Germany<br><br>Dr. med. Benjamin Kühne (Project management)<br>University hospital of Cologne, Department of Pediatric and Adolescent Medicine, Neonatology and Pediatric Intensive Care Medicine, Kerpener Str. 62, 50937 Cologne, Germany
**STUDY CENTER**	University hospital of Cologne, Department of Pediatric and Adolescent Medicine, Neonatology and Pediatric Intensive Care Medicine, Kerpener Str. 62, 50937 Cologne, Germany
**PRIMARY OBJECTIVE**	The influence of extrauterine placental transfusion on the mean hematocrit value within the first 24 hours after birth compared to the standard procedure using DCC.
**STUDY DESIGN**	Randomized, single-center, two-arm parallel, rater-blinded intervention study.
**STUDY ARMS**	1) **Intervention group:** Extrauterine placental transfusion (EPT)<br>2) **Control group:** Delayed cord clamping (DCC)
**SAMPLE SIZE**	60 births (including up to 30% twin pregnancies) in two arms of 30 birth
**INCLUSION CRITERIA**	• Birth weight < 1500 gram ("very low birth weight infant")<br>• Gestational age at birth >23+6 weeks<br>• Delivery by cesarean section<br>• Delivery in the tertiary care perinatal center of the University Hospital Cologne

	• Written consent of the legal guardian
**EXCLUSION CRITERIA**	- Vaginal birth<br>- Fetal or maternal risk (i.e. compromise, emergency c-section)<br>- Congenital anomalies and/or major cardiac defects<br>- Placental abruption or previa with hemorrhage<br>- Placenta accreta or increta<br>- Monochorionic multiples (i.e. Di/Mo or Mo/Mo twins)<br>- Multiples with 3 or more infants<br>- Parents declined study<br>- Severe maternal disease (e.g. COVID-19 infection)
**SCHEDULE**	recruiting first participant: quarter II/2019<br>recruiting last participant: quarter II/2020<br>study completion: quarter III/2022

5 RESPONSIBILITIES

5.1 KEY TRIAL CONTACTS

Priv.-Doz. Dr. med. André Oberthür (Principal Investigator)
University hospital of Cologne, Department of Pediatric and Adolescent Medicine, Neonatology and Pediatric Intensive Care Medicine, [redacted]

Priv.-Doz. Dr. med. Angela Kribs (Co-Principal Investigator)
University hospital of Cologne, Department of Pediatric and Adolescent Medicine, Neonatology and Pediatric Intensive Care Medicine, [redacted]

Dr. med. Benjamin Kühne (Project management)
University hospital of Cologne, Department of Pediatric and Adolescent Medicine, Neonatology and Pediatric Intensive Care Medicine, [redacted]

5.2 CO-INVESTIGATORS

Dr. med. Berthold Grüttner
University of Cologne, Faculty of Medicine and University Hospital Cologne, Department of Gynecology and Obstetrics, [redacted]

Prof. Dr. rer. medic. Martin Hellmich, Dipl.-Math.
University of Cologne, Faculty of Medicine and University Hospital Cologne,
Institute for Medical Statistics and Computational Biology, [redacted]

Jun.-Prof. Dr. Dr. nat. med. Miguel Alejandre Alcazar
University of Cologne, Faculty of Medicine and University Hospital Cologne, Department of Pediatric and Adolescent Medicine, Translational Experimental Pediatrics— Experimental Pulmonology, [redacted]

5.3 STEERING COMMITTEE

A Steering Committee will provide ongoing monitoring of the study, particularly with respect to the benefit-risk analysis with an evaluation of the common complications of preterm birth. The Steering Committee will consists of physicians, nurses, and a statistician:

Prof. em. Dr. Bernd Roth (chair)
Priv.-Doz. Dr. Angela Kribs

Priv.-Doz. Dr. André Oberthür

Dr. Benjamin Kühne

Dr. Barbara Hero

Prof. Dr. Martin Hellmich

Ms. Anne Siegert (Pediatric critical care and anesthesia nurse)

Ms. Sonja Philippi (Pediatric nurse)

5.4 PARTICIPATING INSTITUTIONS / STUDY CENTER / INVESTIGATORS / EXTERNAL STUDY MONITORING

Delivery room and prenatal ward (ward 6)
Department of Gynecology and Obstetrics
University hospital Cologne
Kerpener Str. 34
50931 Cologne

Neonatology and Pediatric Intensive Care Medicine
Department of Pediatric and Adolescent Medicine
University hospital Cologne
Kerpener Str. 62
50937 Cologne

Faculty of Medicine and University Hospital Cologne
Department of Pediatric and Adolescent Medicine
Translational Experimental Pediatrics - Experimental Pulmonology
Kerpener Str. 62
50937 Cologne

Comparative Medicine
Center of Molecular Medicine Cologne (CMM)
University hospital Cologne
Robert Koch Str. 21
50931 Cologne

Social pediatric center
Department of Pediatric and Adolescent Medicine
Kerpener Str. 62
50937 Cologne

Institute for Medical Statistics and Computational Biology
University of Cologne
Bachemer Str. 86

50931 Cologne

Pediatric clinical trial center
Department of Pediatric
University hospital Cologne
Kerpener Str. 62
50937 Cologne

The monocenter trial will be conducted at one site (Neonatology and Pediatric Intensive Care Unit of the Department of Pediatrics and Adolescent Medicine of the University Hospital of Cologne) in cooperation with the delivery room and prenatal unit (Department of Obstetrics and Gynecology), as well as the Social Pediatric Center (SPC) of the Department of Pediatrics and Adolescent Medicine. The experimental part of the study is conducted by the Experimental Pneumology Group of the Department of Pediatrics and Adolescent Medicine and the Center for Molecular Medicine Cologne (ZMMK). Statistical management of the project is provided by the Institute for Medical Statistics and Computational Biology at the University of Cologne. Informed consents will be obtained from physicians of both the obstetrics and neonatology departments.

5.5 TRIAL REGISTER

The study will be registered in the German Register of Clinical Trials (DRKS).

6 BACKGROUND

The fetal-neonatal transition, the transition from intrauterine to extrauterine life, is characterized by a large number of changes in important physiological processes. The main factors are ventilation of the lungs and gas exchange by starting of regular spontaneous breathing, as well as a transition of the circulatory system due to increased pulmonary blood flow and a sequential perfusion of the pulmonary and systemic circulation.

In the fetal-neonatal transition, the timing of clamping the umbilical cord of a newborn from the placental blood circulation represents an important aspect that has become the focus of neonatology research in recent years. On the one hand, it has been shown that delayed cord clamping (DCC), or alternatively cord milking (CM), leads to a higher postnatal blood volume in premature infants, improves cerebral oxygenation in the first minutes of life, and also has a favorable effect on neonatal outcome. Foremost in this context is a significant reduction in the rate of intracerebral hemorrhage (IVH) (1, 18). On the other hand, animal data demonstrate significantly improved hemodynamic stability in the fetal-neonatal transition period when the umbilical cord is not clamped until the lungs are fully aerated (2). This evidence has led to a WHO recommendation in 2012 that DCC should be performed for at least 60 seconds after birth in resuscitation of mature newborns. (3). However, particularly premature "very low birth weight" (VLBW) preterm infants with a birth weight of less than 1500 grams seem to benefit from delayed cord clamping (4, 19).

In clinical practice, the challenge is that DCC or CM usually also delays the initiation of respiratory support (usually mask continuous-positive-airway-pressure (CPAP)) therapy), because no technique has yet been established to initiate CPAP therapy immediately at the bedside next to the mother. Although the use of mobile neonatal resuscitation units that allow bedside respiratory support is currently being investigated clinically (9-11), there are still significant limitations to this approach, for example, due to the relatively short umbilical cord of VLBW preterm infants or in case of multiples, who represent a large proportion of VLBW preterm infants (5). Thus, DCC/CM can disturb the physiologically parallel processes of lung ventilation and increase in preload due to placental transfusion (6). This results in transient changes in systemic blood pressure and perfusion differences in animal models with associated negative effects on cerebral oxygenation (7).

6.1 CLINICAL PART

Several years ago, an alternative procedure to delayed umbilical cord clamping was introduced at the University hospital of Cologne. In this procedure, premature infants are born by caesarean section and the placenta is detached from the uterus at the same time. The infants are then transferred to resuscitation unit with the connection to the placenta still intact, who then perform placental blood transfusion by holding the placenta up about 40-50cm above the infant's heart level, while CPAP respiratory support via mask is started at the same time. This procedure, termed "extrauterine placental transfusion" (EPT), could allow for both the advantages of late weaning and the advantages of adhering

to the physiological sequence of weaning only after lung ventilation has occurred. Currently, this procedure is used at the University Hospital of Cologne as an equivalent procedure to delayed weaning/ umbilical cord expulsion in the majority of cesarean deliveries of VLBW infants.

Although this approach was sporadically described as early as the 1970s and was associated with better outcome of preterm infants at that time (12,13), to our knowledge it is not a common procedure. Similarly, no scientific studies of the procedure are currently found regarding its effects on essential physiological parameters, such as cerebral and systemic oxygenation, postnatal hematocrit, nor have the advantages or disadvantages of this procedure compared with the current standard of DCC/CM procedure been studied in terms of neonatal outcome.

6.2 EXPERIMENTAL PART

Due to the immaturity of the lungs, premature infants frequently develop respiratory distress syndrome initially and, in further course, a chronic lung disease also known as bronchopulmonary dysplasia (BPD). The main characteristic of this disease is a reduced gas exchange surface due to a disturbed formation of alveoli and blood vessels, which in turn can lead to pulmonary arterial hypertension. Therefore, the question arises in particular: How can vascularization and thus lung growth be preserved and induced?

Pulmonary angiogenesis is essential for the formation of alveoli. Experimental work demonstrates that angiogenesis induces secondary septation of the lung. Inhibition of angiogenesis leads to emphysema and pulmonary hypertension; stimulation of angiogenesis, on the other hand, protects against the development of neonatal chronic lung disease and reduced lung growth (26)(27)(28).
DCC/CM increases not only systemic but also pulmonary intravascular volume. As a result, the pressures acting on the endothelial cell as well as shear forces are significantly different. In this regard, there is increasing evidence from experimental studies that mechanical stress in terms of pressure and shear force, influences and even induces angiogenesis (29)(30)(31). Complementary to the mechanical forces on the endothelium, increased growth factors and cytokines may be transmitted through the DCC/CM into the fetal pulmonary circulation. Mechanical growth stimuli coupled with increased concentrations of growth factors and cytokines could favor alveolar formation in preterm infants with DCC/CM.
Placental blood entering the neonatal blood circulation via the umbilical cord is itself rich in cytokines and stem cells, and thus may in turn influence the processes occurring in the pulmonary circulation (24)(25).
Based on our own preliminary work and recent studies, we now want to investigate the hypothesis that delayed umbilical cord clamping promotes lung growth and protects against chronic lung disease by altering volume ratios on the one hand and by increasing circulating growth factors and cytokines on the other hand (32)(33).

6.3 RATIONAL AND JUSTIFICATION FOR THE STUDY

In a retrospective cohort analysis of preterm infants < 32 weeks of gestation in 2015 at the University Hospital of Cologne, Germany, it was observed that infants who received EPT during neonatal resuscitation had a comparable neonatal outcome (8), without any negative effects of the procedure

being observed. Furthermore, the results of this retrospective study are also supported by the annual evaluation of the Institute for Quality Assurance and Transparency in Health Care, which centrally records case numbers and neonatal outcome of preterm infants in Germany. Preterm infants born at the University Hospital of Cologne in 2012-2016 have the highest values for overall survival and survival without severe disability in a nationwide comparison (14). The Cologne model of neonatal resuscitation of preterm infants under 1500g birth weight with the use of EPT thus does not seem to have a negative impact on survival or survival with severe disability of VLBW infants.

However, as a limitation, the retrospective analysis could not include the individual umbilical cord clamping strategy of the control group, i.e. those patients in which the umbilical cord was already cut in the delivery room by the obstetrician. Thus, all cord clamping strategies ("immediate cord clamping" (ICC) or DCC or CM) were included in one group.

To further investigate the effects of the EPT procedure performed in Cologne, EPT will now be compared with a defined cord clamping strategy in a randomized trial.
For this purpose, the cord-clamping strategy of the DCC is to be chosen, primarily because the best scientific evidence currently exists for this in the neonatal resuscitation of VLBW infants. The use of a DCC for 30-180 seconds is explicitly recommended in the current European Resuscitation Council (ERC) guidelines for preterm infants (37) and is also reflected in obstetric guidelines (38). CM as a possible alternative procedure (19, 20) is currently not definitively established in the care of VLBW infants despite increasing evidence.

In addition to the impact on important neonatal outcome parameters already recorded in routine clinical practice, the effects of the two cord clamping procedures on cerebral blood flow during the first minutes of life seem to be particularly relevant. Postnatal cerebral blood flow has an important role in the incidence of IVH, an important neonatal morbidity in VLBW infants. Near-infrared spectroscopy (NIRS) is a noninvasive technique for monitoring regional cerebral oxygen saturation (rScO2), providing information about both cerebral oxygenation and cerebral blood flow. It is an established technique in the monitoring of neonatal resuscitation of preterm infants (16).

7 PROJECT OBJECTIVES

7.1 HYPOTHESIS AND PRIMARY OBJECTIVE

Hypothesis: EPT increases postnatal blood volume and hematocrit in the first 24 hours after birth.
The primary objective of the study is therefore to investigate the influence of EPT on the hematocrit value recorded by routine postnatal blood samplings within the first 24 hours after birth compared to the standard procedure using DCC.

7.2 HYPOTHESIS AND SECONDARY OBJECTIVES

Hypothesis: EPT contributes to stabilization of preterm infants during neonatal resuscitation and provides improvement of neonatal outcome.
Therefore, the secondary aim of the study is to evaluate the influence of EPT on vital parameters and cerebral tissue oxygenation, the occurrence of neonatal morbidities, and long-term neurocognitive outcome compared to DCC.
Furthermore, we will investigate the hypothesis that EPT promotes lung growth and protects against BPD via an altered volume status, as well as via an increase in circulating growth factors and cytokines. The aim of the experimental part of the study is to characterize differences in the concentration distribution of angiogenic factors in serum between EPT and DCC. The second step is to investigate whether serum from placental blood and from children with and without ETP and DCC has a pro-angiogenic effect and stimulates lung vascularization.

7.3 FEASIBILITY

Another aim of the study is to investigate the feasibility of EPT.
According to conservative estimates, in 10-20% of elective cesarean deliveries it is not possible to deliver the placenta together with the infant. Possible reasons for this are, for example, a deep position of the placenta, adhesions and a possible maternal blood loss. In addition, the feasibility of EPT after birth of the infant in the context of neonatal resuscitation will be investigated.
Therefore, another aim of the study is to evaluate the frequency with which EPT is not feasible and to detect possible influencing factors.

8 STUDY OBJECTIVES

8.1 PRIMARY OUTCOME

The mean hematocrit value from blood samples taken within the first 24 hours of life is defined as the primary outcome.
All hematocrit determinations within the first 24 hours of life collected during routine clinical care are used for averaging, if at least one determination was performed.
The measurement of the primary outcome is performed by the central laboratory of the Institute of Clinical Chemistry at the University Hospital of Cologne within the framework of clinically indicated routine blood sampling. The value is given as a percent. Hematocrit is usually determined at birth, after 12 hours and after 24 hours. For the last value at 24 hours, a time variance of up to ±2.5 hours (=10%) is tolerated.

8.2 SECONDARY OUTCOME

In addition to parameters such as singleton/multiple pregnancy, mode of delivery, birth weight, and gestational age, secondary outcome measures include parameters that are routinely collected as part of routine clinical health care. This includes: Data on umbilical cord clamping and respiratory function monitoring parameters, blood values, complications of prematurity, infections, and neurocognitive development of the infants, as well as influencing factors and complications in the mother.
In the experimental part of the study, the serum of the infants will be analyzed at two different time points for the presence of different pro- and anti-inflammatory as well as growth factors and subsequently investigated whether there are differences in the effect on endothelial cell homeostasis of human lung endothelial cells.

8.3 STUDY DESIGN

This will be a randomized, monocentric trial with control and intervention arms and stratification by gestational age and singleton/multiple pregnancy. As far as possible, the assessment of the outcome variables will be blinded.

9 STUDY POPULATION

9.1 INCLUSION CRITERIA

- Birth weight < 1500 gram ("very low birth weight infant")
- Gestational age at birth >23+6 weeks
- Delivery by cesarean section
- Delivery in the tertiary care perinatal center of the University Hospital Cologne
- Written consent of the legal guardian

9.2 EXCLUSION CRITERIA

- Vaginal delivery
- Fetal or maternal risk (i.e. compromise, emergency c-section)
- Congenital anomalies and/or major cardiac defects
- Placental abruption or previa with hemorrhage
- Placenta accreta or increta
- Monochorionic multiples (i.e. Di/Mo or Mo/Mo twins)
- Multiples with 3 or more infants
- Parents declined study
- Severe maternal disease (e.g. COVID-19 infection)

9.3 NUMBER OF PARTICIPANTS

60 births (including approximately 30% twin births) will be included. In the case of twins, for medical reasons, only the second-born infant can be randomized. The first-born infant will receive the control treatment.

9.4 METHODS FOR ASSIGNING PARTICIPANTS TO TREATMENT GROUPS

Participants will be assigned randomly to study group treatments in a 1:1 ratio according to a blocked sequence of computer-generated, pseudorandom codes. Randomization is provided as a set of opaque numbered letters by the Institute of Medical Statistics and Bioinformatics at the University of Cologne. Randomization will be stratified by:

- Gestational age: 1) 24+0 - 27 + 6 SSW, 2) > 27+6 SSW.
- Type of gravidity: 1) singleton, 2) multiple

9.5 SUBSEQUENT EXCLUSION OF STUDY PARTICIPANTS

Initially included infants and mothers may be subsequently excluded from the study if

- the infant does secondarily meet one or more exclusion criteria (e.g., prenatally undiagnosed severe underlying disease),
- the informed consent of the legal guardians is withdrawn.

9.6 PROCEDURE IN CASE OF NON-FEASIBILITY OF THE INTERVENTION

If it is not possible to perform study intervention for medical reasons (despite assignment to the intervention group), the participant remains in the study (intention-to-treat principle).

9.7 APPROACH TO DICHORIAL TWIN PREGNANCY

For obstetric reasons, birth of both twin infants each with their own placenta is not applicable in a majority of dichorial twin birth.
However, if the inclusion criteria of the study are basically fulfilled in case of twin pregnancy, dichorial twins can also be included in the study.
In this case, a delayed cord clamping (DCC) procedure is always performed for twin 1 (defined as the baby that the obstetrician develops first after caesarean section).
Twin 2 is randomized to receive either EPT or DCC depending on the randomization assignment. The firstborn twin will be recorded with respect to the primary outcome measure and, as far as possible, with respect to the secondary outcome measures.
The second-born randomized twin will be assigned to the neonatal resuscitation unit, which will allow measurement of all target criteria, including respiratory function monitor.

9.8 PROCEDURE IN CASE OF MISSING HEMATOCRIT MEASUREMENT

If no hematocrit was determined in the first 24 hours of life (e.g. due to death of the participant within the first hours of life), the other target values of the study participant will still be recorded and evaluated separately.

10 STUDY PROCEDURE

10.1 SCREENING AND ELIGIBILITY ASSESSMENT

The informed consent of the parents or legal guardians is obtained prior to birth at the Department of Gynecology and Obstetrics, according to the inclusion and exclusion criteria. Reasons for an expected premature birth are maternal (e.g. pre-eclampsia, HELLP syndrome, cervical insufficiency, amniotic infection) or fetal (e.g. intrauterine growth retardation, pathological CTG) pregnancy complications. For this purpose, pregnant women > 23+6 weeks' gestation and < 32 + 0 post menstruationem, in whom premature delivery of the infant is expected < 32 + 0 gestational age and in whom the estimated birth weight of the infant is < 1500 grams, will be informed about the study as potential study participants.

The infant will only be included in the study if the legal guardians (usually both parents, in the case of a single parent, the sole legal guardian) have given their consent after they have been informed about the nature, significance and scope of the trial in an appropriate and comprehensible way, by a physician of obstetrics and neonatology, both orally and in writing, and at the same time have declared with their consent that they agree with the recording of data that will take place within the framework of the trial. If there is a twin pregnancy, a signed declaration of consent from the legal guardians must be available for each infant.

The parents are informed that they can withdraw their consent at any time without giving reasons and without any disadvantages for their infant or themselves. The original written consent will be kept in the study folder at the study center. A copy of the written information and informed consent will be given to parents and guardians.

Since a large number of the described complications leading to premature birth can occur in acute and also unexpected ways, prenatal informed consent of both guardians is not always possible (e.g. in case of a business trip of the father). In this case, the second guardian (usually the father) is informed as soon as possible after birth. If consent is then refused, the infant will be excluded from the study.

The original of the information for the legal guardians and the written consent form will be kept in a study folder in the study center.

10.2 PROCEDURE IN CONTROL/INTERVENTION GROUP

The trial consists of 3 periods.

The first period will start shortly before the birth of the infant with a randomized assignment of the infant to the intervention or control group as well as the implementation of EPT immediately after birth

or delayed umbilical cord clamping and extended monitoring of vital parameters during neonatal resuscitation.

The second period comprises the hospital stay and includes the documentation of clinical parameters and blood tests of the infants, as well as of the mothers. This period ends with an individually variable duration at discharge of the infant and the mother.

The third period occurs at a corrected age of 24±2 months and includes the assessment of the BSID III.

10.2.1 FIRST STUDY PERIOD

10.2.1.1 BEFORE THE BIRTH

Medical records of mothers who have been informed about the study and for whom consent to study participation has been obtained from the legal guardians will be labeled on the patient's record ("Participant EXPLAIN Trial"). Obstetrics will inform Neonatology of the mother's impending delivery or the impending delivery of a study participant. The neonatologist and the obstetrician in charge will now make sure that the informed consent form signed by both guardians is available. The sealed randomization envelopes for the study participants are kept in a location accessible and known to the neonatologists in charge at the neonatal intensive care unit and are now opened in his presence of both physicians. Initially, the last name, first name and date of birth of the infant are handwritten on the randomization form, which is later covered with a patient label. This allows the unique assignment of the randomization ID to an infant.

The randomization order is communicated before the start of the cesarean section, to all staff involved in delivery and neonatal resuscitation.

The neonatology staff must ensure that the recording of additional study-related parameters can take place. For this purpose, the extended respiratory function monitor is prepared and a kitchen weighing scale is provided on the resuscitation unit.

10.2.1.2 PROCEDURE FOR TWIN PREGNANCY PRIOR TO BIRTH:

Similar to the procedure described above, the obstetrician and neonatologist must ensure that a signed informed consent form for study participation is available for each infant. A randomization envelope will be opened prior to delivery. The randomization envelope is only valid for **twin 2** at delivery. At the birth of **twin 1**, the procedure of the control group ("delayed cord clamping" (DCC)) described in section 9.7 is always chosen (independent of the randomization envelope for **twin 2**). **Twin 1** is defined as the child that the obstetrician develops first after caesarian section.

Analogous to the procedure described above, the surname, first name, and date of birth of second twin is noted on the randomization letter or later pasted over with patient label.

Before the start of the cesarean section, the randomization notification is communicated to all staff involved in the delivery and neonatal resuscitation of the infant. It must be ensured in advance that the

randomized infant **(= twin 2**) is transferred to the neonatal resuscitation unit with the extended respiratory function monitoring (including NIRS).

10.2.1.3 PROCEDURE IN THE CONTROL GROUP DURING AND AFTER BIRTH

In the control group, delayed cord clamping (DCC) is performed by the obstetrician and neonatologist. As soon as the infant is born, the midwife or neonatologist receives the infant with the umbilical cord still intact and the APGAR clock is started. The infant will be dried and wrapped in warm towels or a sterile plastic bag (neoHelp, Vygon, Aachen, Germany) (for extremely premature infants < 1000 g birth weight). By using gentle tactile stimuli (careful stroking over the back/hands/feet), the infant's spontaneous breathing is stimulated. While performing DCC, the infant is held at the level of the maternal pelvis (=placental level) with the umbilical cord still intact, if possible. The cord is cut after at least 30 seconds, but should not extend beyond 180 seconds.

During this period, obstetrics and neonatology are allowed to stop the DCC procedure and perform an immediate umbilical cord clamping < 30 seconds (=ICC) if one of the following criteria is fulfilled:

Mother	– Hemorrhage, hemodynamic instability, or both.<br>– (not known) placenta praevia, placental abruption
Infant	– Requirement for immediate neonatal resuscitation on neonatal care unit (avital infant with no muscle tone, no spontaneous breathing > 30 seconds).<br>– Interrupted placental blood circulation (placental abruption, placenta praevia)

Table 1: Clinical settings in which immediate umbilical cord clamping (ICC) should be considered in the control group (according to (38)).

After the umbilical cord has been cut by the obstetric physician, the infant is transferred to the neontal resuscitation unit of the neonatology department.

The infant is given initial care in accordance with the clinic's internal standards: The infant is further placed in warm towels or in an occlusive polyethylene wrap (NeoWrap™, Fisher&Paykel Healthcare Limited, Auckland, New Zealand) for temperature management or remains wrapped in the sterile plastic sheet (neoHelp, Vygon, Aachen, Germany), it is placed in the left lateral position (so-called "comfort position") and the neonatologist initiates respiratory support using a CPAP mask according to a standardized internal protocol. At the same time the nurse establishes a standardized monitoring which includes transcutaneously measured blood oxygen saturation, heart rate, respiratory rate and tidal volumes.

As an additional monitoring parameter, regional cerebral oxygen saturation (rScO2) will be measured using near infrared spectroscopy (NIRS). The measurement will be established non-invasively via the additional application of a self-adhesive neonatal NIRS sensor to the midline forehead of the infants. It involves the recording of only one channel (1-channel NIRS), the second channel is not recorded. The

measurement is recorded for the duration of neonatal resuscitation approximately 60 minutes, after which the sensor is removed.

The arrangement in the context of the neonatal resuscitation of the control group is shown in Figure 1.

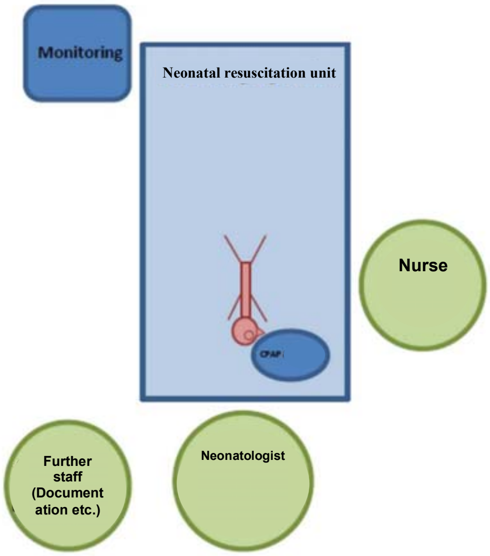

Figure 1: Schematic illustration of initial treatment of infants in the control group

10.2.1.4 PROCEDURE IN THE INTERVENTION GROUP DURING AND AFTER BIRTH

In the intervention group, the infant is delivered by obstetrics together with the placenta. Here, birth occurs either in the intact amniotic sac or, when the amniotic sac is open, by gently detaching the placenta from the uterus. In any case, the infant is transferred with the umbilical cord and placenta still intact by the obstetrician or midwife to the neonatal resuscitation unit of the neonatology department.

If the amniotic sac is still intact, the neonatologist opens it on the neonatal resuscitation unit.

Neonatal resuscitation is also provided according to the internal clinical standards: the infant is covered with an occlusive polyethylene wrap (NeoWrap™, Fisher&Paykel Healthcare Limited, Auckland, New Zealand) for temperature management, the infant is placed in the left lateral position (so-called "comfort position") and the neonatologist initiates respiratory support using a CPAP mask according to a standardized internal protocol. At the same time, the nurse establishes standardized monitoring, which includes transcutaneously measured blood oxygen saturation, heart rate, respiratory rate and respiratory volumes. An NIRS monitoring is also established, analogous to the procedure in the control group.

Additionally, a further staff member (neonatologist 2 or nurse 2 or midwife) stores the placenta in a sterile plastic bag (neoHelp, Vygon, Aachen, Germany) immediately after opening the amniotic sac or after transferring the infant to the neonatal resuscitation unit, where it remains until umbilical cord is clamped. The placenta is sterilely stored and weighed on a separate kitchen scale, then kept at a level of about 40 cm above the infant's umbilicus for EPT. The exact level depends on the length of the

infant's umbilical cord and can therefore vary individually. EPT is to be performed from the time the placenta is held up for a period of at least 1 minute but not more than 10 minutes. After umbilical cord clamping, placenta shall be weighed again on the separately provided calibrated kitchen scale and the weight documented.

The arrangement in the neonatal resuscitation of the intervention group is shown in Figure 2.

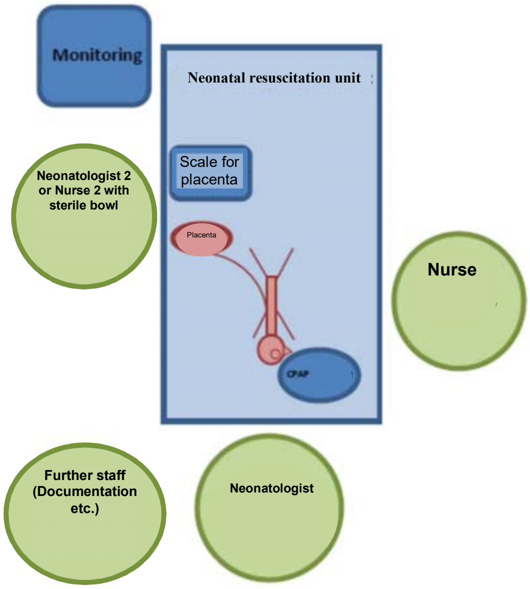

Figure 2: Arrangement in neonatal resuscitation of infants in the intervention group.

10.2.1.5 PROCEDURE FOR TWINS DURING AND AFTER BIRTH

The intervention according to the randomization is only to be performed on **twin 2** (Section 9.7). **Twin 2** is then transferred to the neonatal resuscitation unit where the extended respiratory function monitoring device (with measurement of tidal volume, PEEP, mean airway pressure, cerebral oxygenation) is located. For space limitations, this is usually the left neonatal resuscitation unit in the delivery room of the University Hospital of Cologne.

The firstborn infant (twin 1) is transferred to the right resuscitation unit without extended respiratory function monitoring after birth and DCC or the procedures described in Section 10.2.1.3. The neonatal resuscitation is otherwise analogous to the procedures explained in Section 10.2.1.3.

10.2.1.6 PROCEDURE IF INTERVENTION IS NOT PERFORMED

If, despite allocation of the infant to the intervention group, the development of the placenta by obstetrics is not possible (e.g. deep placental position) or if there are concerns by the obstetrics (long duration of development, maternal complications such as severe blood loss, etc.), it is up to obstetrics to stop the intervention in this situation and, if necessary, to immediate clamp the cord (ICC) or perform umbilical cord milking (CM). In this case, the further procedure of neonatal resuscitation is

based on the procedure of the control group. Nevertheless, the collection of primary and secondary target criteria is performed. (intention-to-treat principle).

10.2.2 SECOND STUDY PERIOD

During the hospital admission, baseline patient data and target neonatal and maternal outcome data are collected as part of the patients' standard medical record.

10.2.2.1 MANAGEMENT WITH THE USE OF RED BLOOD CELL CONCENTRATES

During the infants' admission to the hospital in the second study period, the indication for transfusion of RBCT concentrates is based on the "liberal RBCT thresholds" defined in the "Effects of Transfusion Thresholds on Neurocognitive Outcome of Extremely Low Birth-Weight Infants" (ETTNO)-trial (34):

Time after randomization	Hematocrit, %			
	restrictive RBCT thresholds		liberal RBCT thresholds	
	critical	noncritical	critical	noncritical
3–7 days of age	<34	<28	<41	<35
8–21 days of age	<30	<24	<37	<31
>21 days of age	<27	<21	<34	<28

'Critical' refers to state of health and is defined as the presence of any of the following: (1) requirement of mechanical ventilation (any mode, excluding continuous positive airway pressure), (2) requirement of continuous positive airway pressure with FiO_2 >0.25 for >12 h per 24 h, (3) patent ductus arteriosus requiring therapy, (4) more than 6 apneas that require stimulation per 24 h, or more than 4 desaturations to SpO_2 <60% per 24 h despite methylxanthines and continuous positive airway pressure and (5) acute sepsis or acute necrotizing enterocolitis requiring inotropic or vasopressor support.

Figure 3: Thresholds for infusion of red cell concentrates in the ETTNO study. From: (34)

Figure 3 illustrates the hematocrit thresholds for RBC transfusion. To meet the definition of critical condition, at least one of the following points must be fulfilled:

- Ventilation including non-invasive positive pressure ventilation (DuoPAP ventilation, high frequency oscillation) and invasive ventilation.
- Non-invasive respiratory support with CPAP and FiO2 requirement > 0.25 for more than 12 hours within the last 24 hours
- Concurrent therapy (medical or surgical) to close a persistent ductus arteriosus
- More than 6 apneas requiring stimulation within the past 24 hours or more than 4 desaturations with an SpO2 <60% in 24 hours despite administration of methylxanthines and CPAP therapy
- Acute Sepsis or NEC requiring inotropic or vasopressor support.

10.2.2.2 DIAGNOSIS OF IVH

Cerebral ultrasound examinations are performed independently of the study by the physicians of the Department of Pediatric Radiology of the Institute of Diagnostic and Interventional Radiology, University Hospital Cologne. The examining physician is blinded to the intervention in the delivery room, i.e. does not know which intervention (EPT or DCC) was performed on the infant. The radiological diagnosis is based on the criteria of Papile et al (16).

10.2.2.3 EXPERIMENTAL PART

For the experimental part of the study, analyses of patient blood samples from both study arms will be performed at two time points. For this purpose, blood from the patient's umbilical cord will be collected (0.5 ml) after the patient's cord has been cut, and 0.5 ml of blood will be collected on the patient's 28th day of life (±2 days) as part of a routine blood sampling. After sample collection, this blood is identified with a prepared label giving the patient's randomization ID. The serum will be centrifuged immediately afterwards in the perinatal center and will be stored at -20°C. Further examinations are performed in the Laboratory of Experimental Pneumology of the Department of Pediatrics and Adolescent Medicine or in the Center for Molecular Medicine Cologne (ZMMK). No additional data will be collected or separate blood samples taken as part of the study.

The serum samples of the patients are delivered to the ZMMK (Laboratory: Comparative Medicine, Head: PD Dr. Esther Mahabir-Brenner) following the strict cold storage. A quantitative determination of pro-/anti-inflammatory and angiogenic factors is performed by enzyme-linked immunosorbent assay (ELISA). The Bio-Plex multiplex system (Bio-Plex 200®, Bio-Rad Laboratories, Inc., Hercules, CA, USA) is used for this purpose. The frozen samples are defrosted at room temperature and processed in the multiplex system according to the manufacturer's instructions. After measurement, analysis of the raw data is performed using Bio-Plex ManagerTM software (Bio-Rad). Based on the median fluorescence intensity, the concentration of cytokines for each sample is calculated by the system and given in pg/ml.

If abnormalities are found here, the use of residual serum material collected from the patients is planned. This second step of the analysis provides for an exposure of commercially available human pulmonary microvascular endothelial cells (HPMVEC) (ScienceCell, 6076 Corte Del Cedro, Carlsbad, CA 92011, United States of America) to the patient serum in order to investigate possible influences on the homeostasis of the endothelial cells. The performance of these experiments is dependent on the amount of residual material. This will be done by standardized molecular genetic methods and will primarily involve measurement of cell proliferation by MTT assay (Promega) for which a small amount of residual material is required and/or BrdU assay (Roche) and secondarily "tube formation assay" as well as cell migration.

10.2.3 THIRD STUDY PERIOD

At the corrected age of the child of 24±2 months, neurocognitive development is assessed by using the Bayley's psychomotor developmental index (PDI) and the mental developmental index (MDI) based on the Bayley Scales of Infant Development (BSID III). This examination takes place as part of

the legal outpatient follow-up of VLBW children at the Social Pediatric Center (SPC) of the Department of Pediatrics and Adolescent Medicine and is recorded as part of the medical follow-up.

For this follow-up of infants, it is ensured that the investigator is blinded to the allocation of the infant to intervention or control group in order to avoid bias.

11 OVERALL DURATION OF THE TRIAL

The recruitment period is approximately 12 months (end of first study period), the discharge of the last recruited patient is about 16 months after the start of the study (end of second study period), the trial is completed after developmental neurological examination of the last recruited patient after about 3.5 years.

12 BENEFIT RISK CONSIDERATION

12.1 INDIVIDUAL BENEFITS ASSOCIATED WITH STUDY PARTICIPATION

The aim of the trial is to investigate the influence of EPT on postnatal blood volume and neonatal outcome of preterm infants with a birth weight lower than 1500 grams. If the hypothesis is confirmed, EPT will lead to a higher postnatal blood volume, resulting in a hemodynamic stabilization of the infants in the first days of life. In the positive case, this may result in a reduction of anemia with the need for donor blood transfusions, as well as a lower incidence of, for example, IVH, BPD as typical neonatal complications, and better long-term neurological development. EPT might thus contribute to a better long-term outcome of very small preterm infants.

12.2 RISKS AND EXPOSURES ASSOCIATED WITH STUDY PARTICIPATION

On the other hand, no relevant risks are known from clinical experience and previous retrospective studies. Theoretically, however, it is possible that EPT may cause polyglobulia resulting in hyperviscosity due to the transfusion of umbilical cord blood. Because of the increased accumulation of hemoglobin, there could be subsequent aggravation of neonatal hyperbilirubinemia. However, there was no evidence for this in the retrospective data analysis from 2015 (8).
Since the collection of the additional blood samples required as part of the study is performed only as part of necessary routine blood sampling, additional stress, pain, and irritation to the infant are minimized.
The sample volume required from the blood collection on day 28 of life is 500µl (0.5ml). According to EMEA recommendations, a single study-related blood sample in premature and newborn infants should not exceed 1% of the infant blood volume, and in the case of multiple blood samples, should not exceed 3% of the infant blood volume cumulatively (22). Based on an infant blood volume of 80 (-100) ml/kg, the first benchmark of 1% would be met by drawing 500µl of blood from an infant body weight of at least 625g (0.625kg x 80ml = 50ml blood volume. 0.5ml / 50ml = 1%). The smallest patients included in the study will be the equivalent of 28 weeks' gestation at the time of blood collection. At this time, the mean body weight is approximately 1000g (23). The collection of 500µl of blood therefore amounts to approximately 0.6% of the infant blood volume, thus no relevant impact of the blood collection on the infants can be assumed.

Overall, we therefore conclude that the risk-benefit ratio is positive.

12.3 RECORDING TYPICAL COMPLICATIONS OF PREMATURITY

The secondary outcome criteria include the typical complications of preterm birth. These include:

- Incidence of respiratory distress syndrome requiring:
    - Respiratory support (CPAP, positive pressure ventilation, non-invasive high frequency ventilation, invasive pressure ventilation, invasive high frequency ventilation).
    - Administration of intratracheal surfactant (invasive and less-invasive).
- Pneumothorax
- Pneumoperitoneum

- Incidence of IVH °I - °IV according to the criteria of Papile et al (16).
- Incidence of periventricular leukomalacia (PVL) and/or porencephaly
- Incidence of NEC (surgical or conservative treatment)
- Incidence of SIP (surgical or conservative treatment)
- Incidence of ROP requiring treatment
- Incidence of BPD, based on criteria defined by Walsh et al (17)
- Incidence of hyperviscosity syndrome caused by polyglobulia with impaired organ perfusion, defined as follows:
    - Polyglobulia characterized by a centrifuged hematocrit >65% or an Hb >22 g/dL in a peripheral venous blood draw and (at least one of the following points apply):
        - FiO_2 requirement >0.3 for more than 12 hours without the presence of other causes such as: respiratory distress syndrome, pulmonary hypertension, vitium cordis, pneumothorax, infection.
        - Lactatemia of >3.0 mmol/l from peripheral venous or arterial blood gas analysis without presence of other causes such as: Vitium cordis, hemodynamically relevant persistent ductus arteriosus, metabolic disease, infection, acute hemorrhage, IVH
        - Reduced diuresis of < 0.5 ml/kg body weight per hour without the presence of other causes including: Infection, persistent ductus arteriosus, drug therapy with indomethacin or ibuprofen.

12.4 CRITERIA FOR PREMATURE TERMINATION OF THE STUDY

The principal investigator may decide to stop the study before its completion. The principal investigator will discuss the termination of the study with the Steering Committee.
In this context, the following complications have to be considered with special attention:

1. Clinical compromise due to hyperviscosity syndrome in terms of impaired organ perfusion (see Section 12.3)
2. severe neonatal hyperbilirubinemia requiring treatment (defined by the number of exchange transfusions)
3. Infant death prior to discharge from hospital
4. Maternal death prior to discharge from hospital.

A summary, of the criteria described above for both study arms will be provided every 3 months and presented to the Steering Committee with an external expert.

13 MODIFICATIONS IN THE STUDY PROTOCOL

There is no provision for amending the testing criteria defined in the study protocol. In exceptional cases, however, changes to the testing criteria are possible. These will only be made after agreement between the study management and the local investigators. Changes in the protocol will be submitted to the Ethics Committee of the Medical Faculty of the University of Cologne.

14 STATISTIC

14.1 STATISTICAL AND ANALYTICAL PLAN

Quantitative target values are described with the number of valid and missing values, mean, standard deviation and percentiles (0, 25, 50, 75, 100 (%)), qualitative target values with absolute (n) and relative (%) frequency. In broad aspects, the statistical analysis is based on a descriptive analysis. P-values are to be interpreted two-sided (exceptions are marked), accordingly two-sided confidence intervals (to the 95% level) are given.

14.2 STUDY POPULATION

The primary analytic dataset is the intention-to-treat (ITT) population. This includes all randomized patients evaluated according to their assigned intervention. Only participants subsequently excluded according to section 9.5 are excluded. Intercurrent events are irrelevant for this treatment rule strategy (36).

The secondary evaluation dataset is the per-protocol (PP) population. This includes all patients included in the study who were treated and observed in a substantially protocol-compliant setting.

14.3 PRIMARY TARGET VARIABLE

The primary target variable is the mean haematocrit value in the first 24 hours after birth.

The hematocrit value is given as percentage. If there are > 1 measured values per patient, the arithmetic mean is determined. Group comparison is done by covariance analysis (ANCOVA) with fixed effects: baseline, group, gestational age (24+0 - 27 + 6 SSW vs. > 27+6 SSW) and gravidity (singleton vs. multiples). The influence of missing values is considered and explored via multiple substitutions (sensitivity analysis).

14.4 SECONDARY TARGET VARIABLES

14.4.1 INFLUENCE FACTORS (INFANT)

- singleton or multiple pregnancy
- birthweight in gramm
- gestational age in days
- sex

14.4.2 TARGET VARIABLES (INFANT)

- APGAR (score 0-10)
- Admission temperature (°C)
- Intratracheal surfactant (yes/no)
- Blood transfusion during first 7 days of life (in ml per kg bodyweight)
- Peak IL-6 in 1st 24 h
- Peak CRP in 1st 24 h (mg/l)

- Peak bilirubin
- Peak haemoatocrit until discharge from hospital
- First measured normoblasts in 1st 24 h (given in normoblasts per 100 leukocytes)
- Neonatal complications:
  - Incidence of intubation and time duration of mechanical ventilation
  - Incidence of spontaneous Pneumothorax/Pneumoperitoneum
  - Incidence of any intraventricular hemorrhage (grades 1-4)
  - Incidence of periventricular leukomalacia or porencephaly
  - Incidence of BPD
  - Incidence of NEC with surgery
  - Incidence of spontaneous intestinal perforation (SIP) with surgery
  - Incidence of blood exchange transfusion due to critical hyperbilirubinemia
  - Incidence of retinopathy of prematurity (ROP) with treatment (drugs and/or surgery)
  - Highest ROP grade (1-5)
  - Incidence of plus disease in infants with ROP
- Incidence of cytomegalievirus (CMV) infection, verified by urin polymerase chain reaction (PCR)
- Incidence of infection with CMV requiring antiviral treatment with Valganciclovir and/or Ganciclovir
- Incidence of death in studied infants until discharge
- Bayley Scales of Infant and Toddler Development - Third Edition (Bayley-III) composite score (motor, cognitive, language) with 22-26 month corrected age

14.4.3 TARGET VARIABLES RECORDED IN PARTICIPANTS OF INTERVENTION (EPT) GROUP

- Duration until cord clamping (sec)
- Weight difference placenta before/after EPT (g)
- Feasibility of EPT (Implementation of the intervention according to the randomization yes/no, statement of rationale in case of deviating procedure)

14.4.4 TARGET VARIABLES RECORDED IN PARTICIPANTS OF CONTROL (DCC) GROUP AND FIRST TWIN

- Time to umbilical cord clamping after DCC (sec).

14.4.5 TARGET VARIABLES RECORDED IN SINGLETONS AND SECOND TWIN

- Mean airway pressure at time points 1,2,3,4,5,6,7,8,9,10 minutes (for twins only second twin).
- Mean tidal volume at time points 1,2,3,4,5,6,7,8,9,10 minutes (in twins only second twin).
- Time to onset of spontaneous breathing (in seconds; in twins only second twin).
- Heart rate at time points 1,2,3,4,5,6,7,8,9,10 minutes (in twins only twin no. 2)
- SpO2 at time points 1,2,3,4,5,6,7,8,9,10 minutes (for twins only twin no. 2)

- rScO2 at time points 1,2,3,4,5,6,7,8,9,10,15,20,25,30,35,40,45,50,60 minutes, respectively (in twins only twin no. 2).

14.4.6 INFLUENCE FACTORS (MOTHER)

- Antenatal administration of steroids (yes or no).
- Presence of premature rupture of membranes (in hours)
- Presence of preeclampsia and/or HELLP syndrome (yes/no in each case)
- Highest CRP level in the period 48 hours before birth
- Number of previous pregnancies (not including the current pregnancy)
- Presence of CMV-specific IgM and IgG antibodies in the period ±2 weeks peripartum (in each case yes or no)
- Experience of the obstetrician performing caesarean section (number of caesarean sections performed for preterm infants < 1500 g birth weight: n >100 or n < 100)

14.4.7 TARGET MATERNAL VARIABLES

- Blood loss during labour (in ml).
- Minimal Hb level in 24 hours after delivery (in g/dl)
- Survival until discharge from hospital ("yes" or "no")
- Duration of hospitalization (in days)

14.5 EXPERIMENTAL PART

- Serum analysis by multiplex angiogenesis panel, taken from the umbilical cord after cord clamping:
    - **Pro- and anti-inflammatory cytokines:** IL-1α, IL-1β, IL-6, IL-10, IL-12 (p40), IL-12 (p70), IL-13, IL-17A, granulocyte colony-stimulating factor (G-CSF), granulocyte/macrophage colony-stimulating factor (GM-CSF), monocyte chemotactic protein-1 (MCP-1), macrophage inflammatory protein-1α (MIP-1α), MIP-1β, CXCL10,
    - **Factors of Angiogenese:** sALK-1, amphiregulin, TGFα, epidermal growth factor (EGF), angiopoietin-1/2, sCD31/PECAM-1, endoglin, endothelin-1, basic fibroblast factor (FGF2), hepatocyte growth factor (HGF), leptin, placental growth factor-2 (PLGF-2), prolactin, vascular endothelial growth factor-A (VEGF-A), VEGF-C and VEGF-D, Placental growth factor (PGF), BMP2, BMP4, BMP7, TGFβ, apelin

- Serum analysis of 0.5 ml blood sample collected on day 28 of life (±2 days) as part of a medically indicated blood collection. Analysis by multiplex angiogenesis panel on:
    - **Pro- and anti-inflammatory cytokines:** IL-1α, IL-1β, IL-6, IL-10, IL-12 (p40), IL-12 (p70), IL-13, IL-17A, granulocyte colony-stimulating factor (G-CSF), granulocyte/macrophage colony-stimulating factor (GM-CSF), monocyte chemotactic protein-1 (MCP-1), macrophage inflammatory protein-1α (MIP-1α), MIP-1β, CXCL10,

- **Factors of Angiogenese:** sALK-1, amphiregulin, TGFα, epidermal growth factor (EGF), angiopoietin-1/2, sCD31/PECAM-1, endoglin, endothelin-1, basic fibroblast factor (FGF2), hepatocyte growth factor (HGF), leptin, placental growth factor-2 (PLGF-2), prolactin, vascular endothelial growth factor-A (VEGF-A), VEGF-C and VEGF-D, Placental growth factor (PGF), BMP2, BMP4, BMP7, TGFβ, apelin

Differences and changes in the panel variables are described with fold change (to base 2) and evaluated with distribution-free procedures (Wilcoxon rank sum or Wilcoxon signed rank test). The false discovery rate is controlled at 5% (Benjamini Y, Hochberg Y: Controlling the false discovery rate: a practical and powerful approach to multiple testing. Journal of the Royal Statistic Society 1995; 57: 298-300).

14.6 SUBGROUP ANALYSIS

Subgroup analyses will be performed by gestational age (group 1 23+6 - 27+6 SSW, group 2 > 28+0 SSW), sex, and mode of pregnancy (singleton vs. multiples).

14.7 SAMPLE CALCULATION

Our hypothesis is that EPT increases the averaged hematocrit value in the first 24 hours after birth by (relative) 10% (primary outcome), ie, according to (10) from 52.1±6.6% (Table II, DCC only) to 57.31±7.26%. To detect this difference with the two-sample t test for unequal variances in two-sided error of 1st kind with a power of 80%, a case number of 2 times 29 evaluable patients, i.e. about 60 patients, is required (Stata 15.1, StataCorp LLC, College Station, TX, USA; power twomeans). By accounting for covariates (baseline, gestational age, gravity) in ANCOVA, the power reduction due to any missing values can be compensated for. Furthermore, the nine twins randomized to EPT will be compared to their control siblings, where the paired t-test still achieves a power of 80% with a one-sided error of 1st kind of 10% and a correlation of 0.5 between the two measurements (power pairedmeans).

15 DATA MANAGEMENT

15.1 DATA RECORDING

The sources of audit-relevant data are:

- Documentation sheets for gynecologists and neonatologists to record measurements at birth (EPT or DCC), duration of transfusion (EPT and DCC), weight of placenta before and after cord clamping. Original sheets filled out will be stored in a separate study folder.
- Entries in the patient medical chart, ambulatory patient records, hospital databases "Orbis" or "NeoDat". All original documents remain in the patient medical record.
- Acquisition and local storage of vital parameters and rScO2 in neonatal resuscitation by the respiratory function monitor (NewLifeBox Neo-RSD, Advanced Life Diagnostics UG, Weener, Germany) or the NIRS device "Fore-Sight" (company CASMED, Branford, CT 06405, USA). In addition to being stored on a local data storage medium, these data are also read out in a written form and kept in a folder in the perinatal center. These data are stored according to the rules of good scientific practice for the period of at least 10 years.

15.2 DATA MANAGMENT

Data are recorded electronically by trained study personnel in the REDCap 7.4 database (Vanderbilt University Center, 1211 Medical Center Drive, Nashville, TN 37232, USA). All essential data processing procedures are documented completely, clearly, and verifiably at all times. Only the study team has access to the electronic data.

Upon completion of data entry and reconciliation, the data must be recorded on at least 2 archival DVDs, which must be clearly labeled and securely stored in two separate locations. This ensures that the data are documented in a non-alterable form.

The documentation sheets, electronic data of the respiratory function monitoring and the NIRS measurements, consent documents, as well as other important examination documents are stored for at least 10 years.

15.3 DATA PROTECTION

The requirements of the Data Protection Law are fulfilled. It is ensured that all research materials and data are adequately anonymized in accordance with data protection regulations prior to scientific utilization.

16 BIOMATERIALS MANAGEMENT

In the study, blood is collected at two time points. The first collection of approximately 0.5 ml of whole blood is taken from the umbilical vein of the umbilical cord, after the placenta has been detached from the infant's blood circulation, and thus does not represent a direct intervention on the patient. The second blood sample is taken on the 28th day of life (±2 days) as part of a medically indicated blood sampling. The blood volume is 0.5 ml of whole blood.

Immediately after the blood sampling, the serum is centrifuged by a centrifuge located in the perinatal center, which is used in daily clinical routines for the centrifugation of blood samples. The samples are labeled with prepared stickers using the randomization ID. On the one hand, this makes the blood samples anonymous, on the other hand, it is still possible for the study management to assign the samples to the individual patients. After centrifugation, the serum is immediately stored at -20°C in the freezer of the perinatal center until final use.

Beyond that, no further blood sample collection beyond routine testing will occur.

Placenta and umbilical cord will be transferred to the obstetrics department for further use or disposal as usual after completion of EPT in the intervention group.

17 INSURANCE

The conclusion of a trial insurance for the clinical trial is provided (module 3, category C of the agreement for trial annual insurances for german university hospitals).

18 PUBLICATION POLICY

It is intended to present the results of the study in given time in a scientific journal and/or at german and international congresses. In general, preference should be given to an overall publication. The "Uniform requirements for manuscripts submitted to biomedical journals. International Committee of Medical Journal Editors" [JAMA 1997;277:927-34] will be considered.
For all publications, data security is ensured for all patient data as well as for the data of the participating physicians and other study personnel.

19 REFERENCES

(1) Uwins C, Hutchon DJR: Delayed umbilical cord clamping after childbirth: potential benefits to baby's health. Pediatric Health, Medicine and Therapeutics 2014;5:161-71

(2) Hutchon DJR, Wepster B: The estimated cost of early cord clamping at birth within europe. International Journal of childbirth 2014; Volume 4, Number 4: 250-256

(3) World Health Organization: Guidelines on Basic Newborn Resuscitation, 2012; 7. www.who.int/maternal_child_adolescent/documents/basic_newborn_resuscitation/en/ (last access 15.08.2017)

(4) Hutchon DJ, Mercer JS: Placental transfusion strategies in very preterm neonates: a systematic review and meta-analysis. Obstet Gynecol. 2014 Jul;124(1):47-56

(5) Hutchon D: Evolution of neonatal resuscitation with intact placental circulation. Infant 2014;10(2):58-61

(6) Hooper SB, Polglase GR, Roehr CC 2015 Cardiopulmonary changes with aeration of the newborn lung. Paediatr Respir Rev 16:147-150.

(7) Bhatt S, Polglase GR, Wallace EM, Te Pas AB, Hooper SB: Ventilation before Umbilical Cord Clamping Improves the Physiological Transition at Birth. Front Pediatr. 2014 Oct 20;2:113

(8) Kuehne B, Kirchgaessner C, Becker I, Kuckelkorn M, Valter M, Kribs A, Oberthuer A, Mask Continuous Positive Airway Pressure Therapy with Simultaneous Extrauterine Placental Transfusion for Resuscitation of Preterm Infants - A Preliminary Study. Biomed Hub 2018;3: 488926. doi: 10.1159/000488926

(9) Duley L, Dorling J, Pushpa-Rajah A, Oddie SJ, Yoxall CW, Schoonakker B, Bradshaw L, Mitchell EJ, Fawke JA: Randomised trial of cord clamping and initial stabilisation at very preterm birth. Arch Dis Child Fetal Neonatal Ed. 2018 Jan;103(1):F6-F14. doi: 10.1136

(10) Katheria A, Poeltler D, Durham J, Steen J, Rich W, Arnell K, Maldonado M, Cousins L, Finer N.: Neonatal Resuscitation with an Intact Cord: A Randomized Clinical Trial. J Pediatr. 2016 Nov;178:75-80.e3. doi: 10.1016

(11) Winter J, Kattwinkel J, Chisholm C, Blackman A, Wilson S, Fairchild K.: Ventilation of Preterm Infants during Delayed Cord Clamping (VentFirst): A Pilot Study of Feasibility and Safety. Am J Perinatol. 2017 Jan;34(2):111-116. doi: 10.1055

(12) Dunn PM 1976 Premature delivery and the preterm infant. Ir Med J 69:246-254.

(13) Várdi P Placental Transfusion An Attempt At Physiological Delivery. The Lancet 286:12-13.

(14) Institut für Qualitätssicherung und Transparenz im Gesundheitswesen: Detailergebnisse des Krankenhauses Uniklinik Köln, Tabellen zur Ergebnisqualitätsdarstellung gemäß § 7 Abs.1, 2012-2016. https://perinatalzentren.org/khtable.php?idAE=363 (last access 10.01.2018)

(15) Alderliesten T, Dix L, Baerts W, Caicedo A, van Huffel S, Naulaers G, Groenendaal F, van Bel F, Lemmers P: Reference values of regional cerebral oxygen saturation during the first 3 days of life in preterm neonates. Pediatr Res. 2016 Jan;79(1-1):55-64

(16) Papile LA, Burstein J, Burstein R, Koffler H 1978 Incidence and evolution of subependymal and intraventricular hemorrhage: a study of infants with birth weights less than 1,500 gm. J Pediatr 92:529-534.

(17) Walsh MC, Yao Q, Gettner P, Hale E, Collins M, Hensman A, Everette R, Peters N, Miller N, Muran G, Auten K, Newman N, Rowan G, Grisby C, Arnell K, Miller L, Ball B, McDavid G, National Institute of Child H, Human Development Neonatal Research N 2004 Impact of a physiologic definition on bronchopulmonary dysplasia rates. Pediatrics 114:1305-1311.

(18) Mercer JS, Vohr BR, McGrath MM, et al. Delayed cord clamping in very preterm infants reduces the incidence of intraventricular hemorrhage and late-onset sepsis: a randomized, controlled trial. Pediatrics 2006;117:1235–42.

(19) March MI, Hacker MR, Parson AW, et al. The effects of umbilical cord milking in extremely preterm infants: a randomized controlled trial. J Perinatol 2013;33:763–7.

(20) Katheria A, Garey D, Truong G, Akshoomoff N, Steen J, Maldonado M, Poeltler D, Harbert MJ, Vaucher YE, Finer N. A Randomized Clinical Trial of Umbilical Cord Milking vs Delayed Cord Clamping in Preterm Infants: Neurodevelopmental Outcomes at 22-26 Months of Corrected Age. J Pediatr. 2017 Dec 7

(21) Alvira CM. Aberrant Pulmonary Vascular Growth and Remodeling in Bronchopulmonary Dysplasia. Front Med (Lausanne). 2016; 3: 21.

(22) European Medicines Agency. Guideline on the investigation of medicinal products in the term and preterm neonate. http://www.ema.europa.eu/docs/en_GB/document_library/Scientific_guideline/2009/09/WC500003750.pdf (last access: 04.04.2018)

(23) Fenton TR, Kim JH. A systematic review and meta-analysis to revise the Fenton growth chart for preterm infants. BMC Pediatr. 2013 Apr 20;13:59. doi: 10.1186/1471-2431-13-59.

(24) Takahashi N, Uehara R, Kobayashi M, Yada Y, Koike Y, Kawamata R, Odaka J, Honma Y, Momoi MY. Cytokine profiles of seventeen cytokines, growth factors and chemokines in cord blood and its relation to perinatal clinical findings. Cytokine. 2010 Mar;49(3):331-7. doi: 10.1016/j.cyto.2009.11.024.

(25) Pereira T, Ivanova G, Caseiro AR, Barbosa P, Bártolo PJ, Santos JD, Luís AL, Maurício AC. MSCs conditioned media and umbilical cord blood plasma metabolomics and composition. PLoS One. 2014 Nov 25;9(11):e113769

(26) Le Cras TD, Markham NE, Tuder RM, Voelkel NF, Abman SH. Treatment of newborn rats with a VEGF receptor inhibitor causes pulmonary hypertension and abnormal lung structure. Am J Physiol Lung Cell Mol Physiol. 2002 Sep;283(3):L555-62.

(27) Kasahara Y, Tuder RM, Taraseviciene-Stewart L, Le Cras TD, Abman S, Hirth PK, Waltenberger J, Voelkel NF. Inhibition of VEGF receptors causes lung cell apoptosis and emphysema. J Clin Invest. 2000 Dec;106(11):1311-9.

(28) Thébaud B, Ladha F, Michelakis ED, Sawicka M, Thurston G, Eaton F, Hashimoto K, Harry G, Haromy A, Korbutt G, Archer SL. Vascular endothelial growth factor gene therapy increases survival, promotes lung angiogenesis, and prevents alveolar damage in hyperoxia-induced lung injury: evidence that angiogenesis participates in alveolarization. Circulation. 2005 Oct 18;112(16):2477-86.

(29) Zheng W, Christensen LP, Tomanek RJ. Differential effects of cyclic and static stretch on coronary microvascular endothelial cell receptors and vasculogenic/angiogenic responses. Am J Physiol Heart Circ Physiol. 2008 Aug;295(2):H794-800.

(30) Wilkins JR, Pike DB, Gibson CC, Li L, Shiu YT. The interplay of cyclic stretch and vascular endothelial growth factor in regulating the initial steps for angiogenesis. Biotechnol Prog. 2015 Jan-Feb;31(1):248-57.

(31) Dorland YL, Huveneers S. Cell-cell junctional mechanotransduction in endothelial remodeling. Cell Mol Life Sci. 2017 Jan;74(2):279-292.

(32) Hilgendorff A, Parai K, Ertsey R, Navarro E, Jain N, Carandang F, Peterson J, Mokres L, Milla C, Preuss S, Alcazar MA, Khan S, Masumi J, Ferreira-Tojais N, Mujahid S, Starcher B, Rabinovitch M, Bland R. Lung matrix and vascular remodeling in mechanically ventilated elastin haploinsufficient newborn mice. Am J Physiol Lung Cell Mol Physiol. 2015 Mar 1;308(5):L464-78.

(33) Alejandre-Alcázar MA, Kwapiszewska G, Reiss I, Amarie OV, Marsh LM, Sevilla-Pérez J, Wygrecka M, Eul B, Köbrich S, Hesse M, Schermuly RT, Seeger W, Eickelberg O, Morty RE. Hyperoxia modulates TGF-beta/BMP signaling in a mouse model of bronchopulmonary dysplasia. Am J Physiol Lung Cell Mol Physiol. 2007 Feb;292(2):L537-49.

(34) ETTNO Investigators. The ‘Effects of Transfusion Thresholds on Neurocognitive Outcome of Extremely Low Birth-Weight Infants (ETTNO)’ Study: Background, Aims, and Study Protocol. Neonatology 2012;101:301-305

(35) An International Committee for the Classification of Retinopathy of Prematurity (2005) The international classification of retinopathy of prematurity revisited. Arch Ophthalmol 123:991–999

(36) International Conference on Harmonization (ICH): Estimands and Sensitivity Analysis in Clinical Trials E9 (R1). Current Step 2 version dated 16 June 2017. http://www.ich.org/fileadmin/Public_Web_Site/ICH_Products/Guidelines/Efficacy/E9/E9-R1EWG_Step2_Guideline_2017_0616.pdf (last access: 06.07.2018)

(37) Wylliea J, Bruinenber J, Roehr CC, Rüdiger M, Trevisanuto D, Urlesberger B. European Resuscitation Council Guidelines for Resuscitation 2015 Section 7. Resuscitation and support of transition of babies at birth. Resuscitation 95 (2015) 249–263.

(38) Committee on Obstetric Practice. Committee Opinion No. 684: Delayed Umbilical Cord Clamping After Birth. Obstet Gynecol. 2017 Jan;129(1):e5-e10.
