# Supplementary material for: Extrauterine Placental Perfusion and Oxygenation in Infants With Very Low Birth Weight: A Randomized Clinical Trial
Source: JAMA Netw Open. 2023 Nov 3;6(11):e2340597. doi: 10.1001/jamanetworkopen.2023.40597 (PMC10625045; doi:10.1001/jamanetworkopen.2023.40597)
Supplement: Supplement 2. — eFigure. Umbilical Cord Management for Infants of the Control and Intervention Group eTable 1. Heart Rate and Pulse Oxygen Saturation During Neonatal Transition (Intention-to-Treat Analysis) eTable 2. Cerebral Regional Oxygenation During Neonatal Transition (Intention-to-Treat Analysis) eTable 3. Airway Pressure and Fraction Of Inspired Oxygen Levels During Neonatal Transition (Intention-to-Treat Analysis) eTable 4. Neonatal and Maternal Outcomes (Per-Protocol Analysis) eTable 5. Heart Rate and Pulse Oxygen Saturation During Neonatal Transition (Per-Protocol Analysis) eTable 6. Cerebral Regional Oxygenation During Neonatal Transition (Per-Protocol Analysis) eTable 7. Airway Pressure and Fraction Of Inspired Oxygen Levels During Neonatal Transition (Per-Protocol Analysis) [file jamanetwopen-e2340597-s002.pdf]

Supplemental Online Content

Kuehne B, Grüttner B, Hellmich M, Hero B, Kribs A, Oberthuer A. Extrauterine placental perfusion and oxygenation in infants with very low birth weight: a randomized clinical trial. *JAMA Netw Open*. 2023;6(11):e2340597. doi:10.1001/jamanetworkopen.2023.40597

eFigure. Umbilical Cord Management for Infants of the Control and Intervention Group

eTable 1. Heart Rate and Pulse Oxygen Saturation During Neonatal Transition (Intention-to-Treat Analysis)

eTable 2. Cerebral Regional Oxygenation During Neonatal Transition (Intention-to-Treat Analysis)

eTable 3. Airway Pressure and Fraction Of Inspired Oxygen Levels During Neonatal Transition (Intention-to-Treat Analysis)

eTable 4. Neonatal and Maternal Outcomes (Per-Protocol Analysis)

eTable 5. Heart Rate and Pulse Oxygen Saturation During Neonatal Transition (Per-Protocol Analysis)

eTable 6. Cerebral Regional Oxygenation During Neonatal Transition (Per-Protocol Analysis)

eTable 7. Airway Pressure and Fraction Of Inspired Oxygen Levels During Neonatal Transition (Per-Protocol Analysis)

This supplemental material has been provided by the authors to give readers additional information about their work.

© 2023 Kuehne B et al. *JAMA Network Open.*

eFigure 1: Umbilical cord management for infants of the control (a) and intervention group (b).

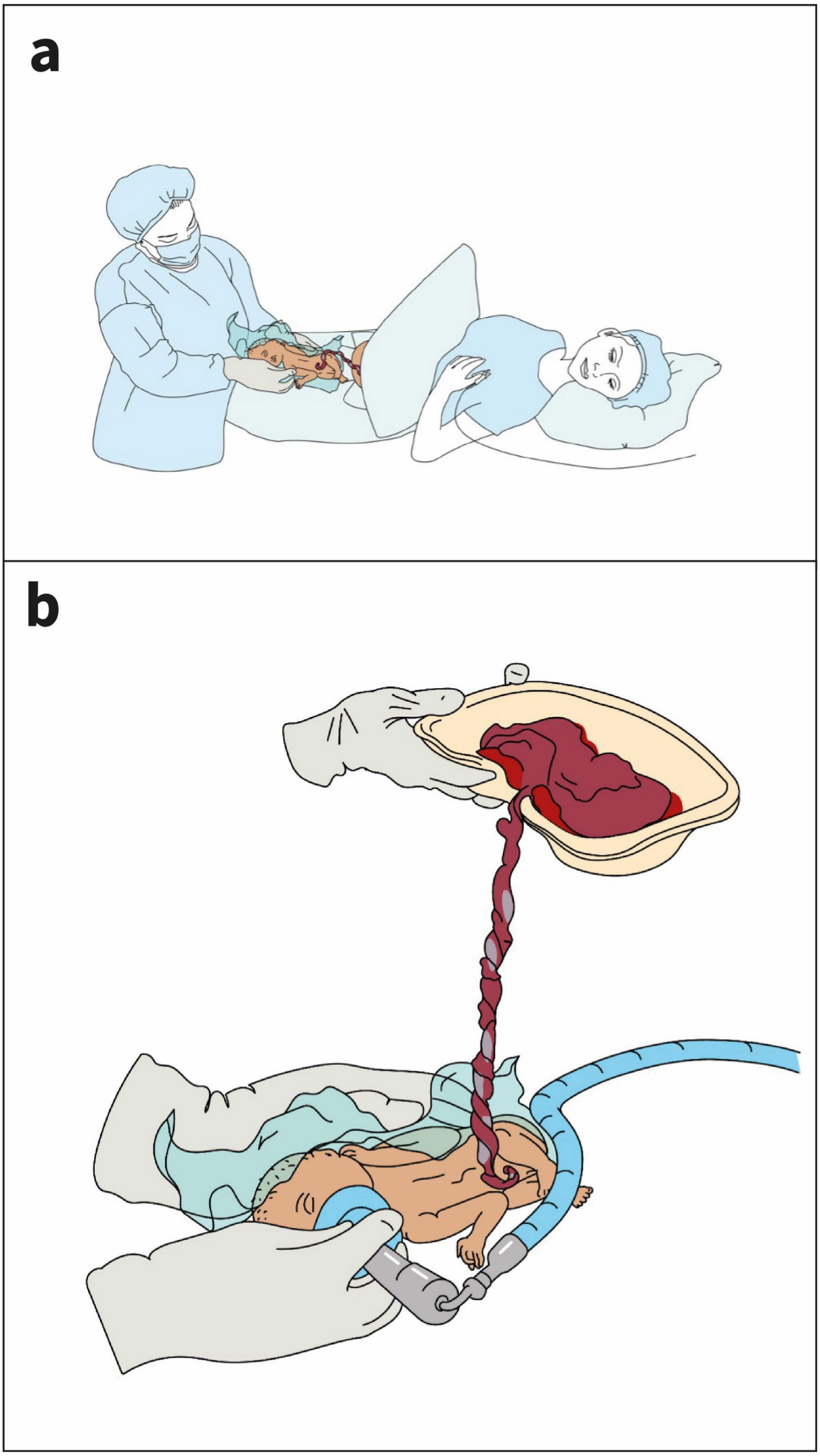

© 2023 Kuehne B et al. *JAMA Network Open.*

eTable 1: Heart Rate and Pulse Oxygen Saturation During Neonatal Transition[a]

		EPP group	DCC group	AMD (95% CI)[b]
Heart rate (bpm) 1 min. after birth, mean ± SD, (n)		108.9 ± 37.2, (9)	N/A	N/A
Heart rate (bpm) 2 min. after birth, mean ± SD, (n)		122.9 ± 35.1, (19)	106.3 ± 39.2, (12)	19.3 (-8.4 to 47.1)
Heart rate (bpm) 3 min. after birth, mean ± SD, (n)		128.6 ± 25.8, (19)	122.3 ± 35.7, (23)	8.6 (-9.9 to 27.2)
Heart rate (bpm) 4 min. after birth, mean ± SD, (n)		126.1 ± 27.5, (19)	132.0 ± 25.6, (25)	-5.4 (-22.1 to 11.4)
Heart rate (bpm) 5 min. after birth, mean ± SD, (n)		134.9 ± 18.9, (18)	133.7 ± 21.7, (26)	1.9 (-10.6 to 14.4)
Heart rate (bpm) 6 min. after birth, mean ± SD, (n)		135.2 ± 24.7, (19)	139.2 ± 19.8, (27)	-2.7 (-16.0 to 10.7)
Heart rate (bpm) 7 min. after birth, mean ± SD, (n)		145.6 ± 16.4, (19)	142.2 ± 20.5, (27)	4.6 (-7.0 to 16.1)
Heart rate (bpm) 8 min. after birth, mean ± SD, (n)		151.0 ± 15.0, (19)	146.7 ± 19.0, (27)	5.5 (-5.0 to 16.0)
Heart rate (bpm) 9 min. after birth, mean ± SD, (n)		151.2 ± 20.5, (19)	151.0 ± 18.2, (27)	1.7 (-9.8 to 13.2)
Heart rate (bpm) 10 min. after mean ± SD, (n)		156.3 ± 15.7, (20)	154.7 ± 16.3, (26)	2.1 (-7.7 to 12.0)
SpO_2 (%) 1 min. after birth, mean ± SD, (n)		62.2 ± 22.3, (9)	N/A	N/A
SpO_2 (%) 2 min. after birth, mean ± SD, (n)		56.9 ± 22.8, (18)	50.3 ± 16.3, (12)	8.8 (-7.4 to 25.0)
SpO_2 (%) 3 min. after birth, mean ± SD, (n)		60.1 ± 25.5, (19)	45.7 ± 19.8, (23)	16.1 (2.3 to 30.0)
SpO_2 (%) 4 min. after birth, mean ± SD, (n)		62.7 ± 26.6, (19)	51.8 ± 20.8, (25)	11.6 (-3.1 to 26.4)
SpO_2 (%) 5 min. after birth, mean ± SD, (n)		70.1 ± 26.1, (19)	55.7 ± 17.9, (26)	15.3 (2.0 to 28.6)
SpO_2 (%) 6 min. after birth, mean ± SD, (n)		77.4 ± 27.3, (19)	68.1 ± 19.7, (26)	11.4 (-2.3 to 25.0)
SpO_2 (%) 7 min. after birth, mean ± SD, (n)		84.2 ± 17.8, (19)	74.6 ± 20.6, (26)	10.8 (-0.6 to 22.2)
SpO_2 (%) 8 min. after birth, mean ± SD, (n)		88.9 ± 10.4, (19)	78.3 ± 17.8, (26)	11.3 (2.0 to 20.6)
SpO_2 (%) 9 min. after birth, mean ± SD, (n)		90.0 ± 11.9, (19)	82.1 ± 15.3, (27)	9.0 (0.3 to 17.6)
SpO_2 (%) 10 min. after birth, mean ± SD, (n)		90.9 ± 8.0, (20)	86.2 ± 13.2, (26)	5.0 (-1.9 to 11.8)

Abbreviations: AMD, adjusted mean difference; bpm, beats per minute; CI, confidence interval; DCC, delayed cord clamping; EPP, extrauterine placental perfusion; NA, not applicable; SD, standard deviation; SpO2, pulse oxygen saturation.

[a] Intention-to-treat set.

[b]AMDs (with 95% CIs) were estimated by univariate analysis of variance, with cofactors (gestational age: 24 weeks 0 days to 27 weeks 6 days and >27 weeks 6 days and type of pregnancy: singleton and multiple).

© 2023 Kuehne B et al. *JAMA Network Open.*

eTable 2: Cerebral Regional Oxygenation During Neonatal Transition[a]

		EPP group	DCC group	AMD (95% CI)[b]
rcSO2 (%) 1 min. after birth, mean ± SD, (n)		52.9 ± 14.1, (11)	37 ± 0, (1)	0.1 (-53.2 to 53.5)
rcSO2 (%) 2 min. after birth, mean ± SD, (n)		53.2 ± 15.0, (17)	49.8 ± 20.7, (18)	2.5 (-10.4 to 15.6)
rcSO2 (%) 3 min. after birth, mean ± SD, (n)		54.6 ± 13.9, (19)	49.4 ± 18.8, (29)	4.6 (-6.0 to 15.3)
rcSO2 (%) 4 min. after birth, mean ± SD, (n)		62.0 ± 15.0, (19)	50.3 ± 15.5, (22)	11.6 (1.7 to 21.5)
rcSO2 (%) 5 min. after birth, mean ± SD, (n)		65.3 ± 15.6, (19)	51.9 ± 17.1, (21)	13.2 (2.4 to 24.0)
rcSO2 (%) 6 min. after birth, mean ± SD, (n)		74.4 ± 13.6, (19)	56.5 ± 17.8, (23)	17.6 (7.4 to 27.7)
rcSO2 (%) 7 min. after birth, mean ± SD, (n)		78.5 ± 9.0, (19)	62.8 ± 15.7, (25)	15.4 (7.1 to 23.6)
rcSO2 (%) 8 min. after birth, mean ± SD, (n)		76.3 ± 14.5, (20)	65.1 ± 15.9, (25)	10.9 (1.5 to 20.3)
rcSO2 (%) 9 min. after birth, mean ± SD, (n)		81.4 ± 11.4, (20)	71.3 ± 14.3, (25)	9.9 (2.0 to 17.8)
rcSO2 (%) 10 min. after birth, mean ± SD, (n)		81.2 ± 10.6, (20)	74.6 ± 14.7, (24)	6.5 (-1.5 to 14.4)

Abbreviations: AMD, adjusted mean difference; CI, confidence interval; DCC, delayed cord clamping; EPP, extrauterine placental perfusion; NA, not applicable; rcSO2, regional cerebral oxygen saturation; SD, standard deviation.

[a] Intention-to-treat set.

[b]AMDs (with 95% CIs) were estimated by univariate analysis of variance, with cofactors (gestational age: 24 weeks 0 days to 27 weeks 6 days and >27 weeks 6 days and type of pregnancy: singleton and multiple).

© 2023 Kuehne B et al. *JAMA Network Open.*

eTable 3: Airway Pressure and Fraction Of Inspired Oxygen Levels During Neonatal Transition[a]

		EPP group	DCC group	AMD (95% CI)[b]
Airway pressure (cmH2O) 1 min. after birth, mean ± SD, (n)		13.2 ± 4.3, (18)	12 ± 4.2, (2)	1.8 (-4.8 to 8.3)
Airway pressure (cmH2O) 2 min. after birth, mean ± SD, (n)		20.7 ± 4.3, (19)	12.6 ± 4.0, (18)	8.7 (6.2 to 11.2)
Airway pressure (cmH2O) 3 min. after birth, mean ± SD, (n)		23.3 ± 4.2, (20)	17.8 ± 6.0, (18)	5.9 (2.5 to 9.3)
Airway pressure (cmH2O) 4 min. after birth, mean ± SD, (n)		24.4 ± 4.4, (20)	20.7 ± 6.3, (18)	4.0 (0.4 to 7.6)
Airway pressure (cmH2O) 5 min. after birth, mean ± SD, (n)		24.7 ± 3.9, (20)	22.5 ± 4.1, (18)	2.2 (-0.5 to 5.0)
Airway pressure (cmH2O) 6 min. after birth, mean ± SD, (n)		24.8 ± 4.3, (20)	22.6 ± 3.9, (18)	2.2 (-0.6 to 5.0)
Airway pressure (cmH2O) 7 min. after birth, mean ± SD, (n)		25.1 ± 4.3, (20)	22.8 ± 3.7, (18)	2.1 (-0.6 to 4.8)
Airway pressure (cmH2O) 8 min. after birth, mean ± SD, (n)		25.2 ± 4.2, (20)	23.3 ± 3.6, (18)	1.8 (-0.8 to 4.5)
Airway pressure (cmH2O) 9 min. after birth, mean ± SD, (n)		24.9 ± 5.0, (19)	23.3 ± 3.5, (18)	1.4 (-1.6 to 4.3)
Airway pressure (cmH2O) 10 min. after birth, mean ± SD, (n)		24.9 ± 4.7, (20)	23.8 ± 3.3, (17)	0.8 (-1.9 to 3.6)
FiO_2 (%) 1 min. after birth, mean ± SD, (n)		26.9 ± 4.9, (14)	21.0 ± 0, (22)	-5.8 (3.7 to 7.9)
FiO_2 (%) 2 min. after birth, mean ± SD, (n)		28.5 ± 5.1, (14)	30.8 ± 8.5, (20)	-2.3 (-7.5 to 2.9)
FiO_2 (%) 3 min. after birth, mean ± SD, (n)		29.7 ± 6.3, (14)	34.4 ± 9.6, (20)	-4.7 (-10.7 to 1.3)
FiO_2 (%) 4 min. after birth, mean ± SD, (n)		34.7 ± 8.5, (14)	37.2 ± 9.8, (20)	-2.5 (9.1 to 4.2)
FiO_2 (%) 5 min. after birth, mean ± SD, (n)		38.1 ± 10.3, (14)	42.7 ± 16.0, (20)	-4.7 (-14.9 to 5.5)
FiO_2 (%) 6 min. after birth, mean ± SD, (n)		37.7 ± 8.9, (14)	44.2 ± 19.8, (20)	-6.5 (-17.9 to 5.0)
FiO_2 (%) 7 min. after birth, mean ± SD, (n)		36.6 ± 8.0, (14)	43.1 ± 21.4, (20)	-6.5 (-18.7 to 5.7)
FiO_2 (%) 8 min. after birth, mean ± SD, (n)		35.5 ± 8.9, (14)	42.4 ± 21.8, (20)	-6.9 (-19.5 to 5.8)
FiO_2 (%) 9 min. after birth, mean ± SD, (n)		33.8 ± 9.8, (13)	43.6 ± 23.0, (20)	-10.2 (-23.9 to 3.6)
FiO_2 (%) 10 min. after birth, mean ± SD, (n)		32.7 ± 11.2, (14)	41.2 ± 23.4, (19)	-8.7 (-22.5 to 5.1)

Abbreviations: AMD, adjusted mean difference; CI, confidence interval; DCC, delayed cord clamping; EPP, extrauterine placental perfusion; FiO2, fraction of inspired oxygen; NA, not applicable; SD, standard deviation;

[a] Intention-to-treat set.

[b]AMDs (with 95% CIs) were estimated by univariate analysis of variance, with cofactors (gestational age: 24 weeks 0 days to 27 weeks 6 days and >27 weeks 6 days and type of pregnancy: singleton and multiple).

© 2023 Kuehne B et al. *JAMA Network Open.*

eTable 4: Neonatal and Maternal Outcomes[a]

Outcome	Participants, No. (%) (N=56)		*AMD or RR (95% CI)[b]*
	**EPP (n=27)**	**DCC (n=29)**	
Apgar score, median (IQR)			
5 min	7 (7 - 8)	8 (7 - 8)	AMD -0.2 (-0.7 to 0.3)
10 min	8 (8 - 9)	9 (8 - 9)	AMD -0.3 (-0.6 to 0.1)
Time to onset of breathing,			
Total with data, No.	24	19	N/A
Mean (SD), sec	9.0 (9.1)	13.3 (24.0)	AMD -4.7 (-13.9 to 4.5)
Time of umbilical cord clamping, mean (SD), sec	484.2 (169.8)	39.0 (8.2)	AMD 445.8 (381 to 510.5)
Weight difference placenta before vs after EPP			
Total with data, No.	23	N/A	N/A
Mean (SD), g	19.9 (9.7)	N/A	N/A
Respiratory support in delivery room			N/A
CPAP	27 (100)	29 (100)	N/A
NIPPV	0	0	N/A
Intubation and mechanical ventilation	0	0	N/A
Surfactant given in delivery room	26 (96.3)	26 (89.7)	0.4 (0.0 to 3.2)
Admission Temperature, mean (SD), °C	36.5 (0.7)	36.3 (0.8)	AMD 0.2 (-0.2 to 0.6)
Mean Hct during first 24 h after birth, mean (SD), percentage points	57.1 (9.4)	54.3 (6.7)	AMD 2.8 (-1.6 to 7.15)
Blood transfusion during first 7 days of life	1 (3.7)	1 (3.4)	1.0 (0.9 to 1.1)
Peak levels			
IL-6 in first 24 h, median (IQR), ng/L	22.0 (11.5 - 45.0)	6.5 (4.0 - 25.0)	AMD 207.4 (-214.8 to 629.6)
CRP in first 24 h, median (IQR), mg/dL	0.16 (0.1 - 0.2)	0.9 (0.1 – 2.0)	AMD 1.4 (-1.5 to 4.4)
Peak Bilirubin in 1st 14 days, mean (SD), mg/dL	10.0 (2.4)	9.9 (2.5)	AMD 0.0 (-1.3 to 1.3)
Duration of Phototherapy, mean ±SD, d	4.8 ± 2.7	4.8 ± 2.3	AMD -0.2 (-1.5 to 1.2)
Exchange transfusion due to severe Hyperbilirubinemia, No. (%)	0	0	N/A
Intubation			
In first 72 h	3 (11.1)	2 (6.9)	1.0 (0.8 to 1.2)
Until discharge	6 (22.2)	5 (13.8)	0.9 (0.7 to 1.2)
Duration of mechanical ventilation, median (IQR), h	318.5 (219.0 - 912.0)	251.0 (172.5 - 379.5)	AMD 291.1 (-447.5 to 1029.7)
Pneumothorax			
In first 7 days of life	1 (3.7)	2 (6.9)	1.0 (0.9 to 1.2)
Until discharge	1 (3.7)	2 (3.7)	1.0 (0.9 to 1.2)
Spontaneous intestinal perforation			
Without surgery	0	2 (6.9)	1.1 (1.1 to 1.2)
With surgery	2 (7.4)	3 (10.3)	1.0 (0.9 to 1.2)
Necrotizing enterocolitis			
Without surgery	0	0	N/A
With surgery	2 (7.4)	0	0.9 (0.8 to 1.0)
Intraventricular Hemorrhage			
Grade 1	4 (14.8)	6 (20.7)	1.1 (0.8 to 1.4)
Grade 2	1 (3.7)	0	1.0 (0.9 to 1.0)
Grade ≥3	2 (3.7)	0	0.9 (0.8 to 1.0)
Periventricular Leukomalacia			
Total with data, No.	26	29	N/A
Yes	0	0	N/A
Bronchopulmonary dysplasia at 36 wk corrected			
Total with data, No.	24	29	N/A

© 2023 Kuehne B et al. *JAMA Network Open.*

Mild	16 (66.7)	15 (51.7)	0.6 (0.3 to 1.3)
Moderate	0	1 (3.4)	1.2 (1.0 to 1.4)
Severe	1 (4.2)	0	0.9 (0.7 to 1.1)
Retinopathy necessitating intervention (medical or surgical)			N/A
Total with data, No.	26	29	N/A
Yes	1 (3.8)	1 (3.4)	1.0 (0.9 to 1.1)
Survival until discharge	27 (96.3)	29 (100)	1.0 (0.9 to 1.0)
Lowest maternal Hb in first 24 h after delivery, mean (SD), g/dL	10.2 (1.1)	10.5 (1.6)	AMD -0.2 (-0.9 to 0.5)
Maternal blood loss during cesarean surgery			
Total with data, No.	25	25	N/A
Mean (SD), mL	460.0 (168.9)	410.0 (252.1)	AMD 63.6 (-53.9 to 181.1)
Maternal postpartum haemorrhage > 1000 mL			
Total with data, No.	25	25	N/A
Yes	1 (4.0)	1 (4.0)	1.0 (0.9 to 1.1)
Chorioamnionitis with maternal CRP > 2.0 mg/dL	3 (11.1)	1 (3.4)	0.9 (0.8 to 1.1)
Maternal survival until discharge	27 (100)	29 (100)	N/A

Abbreviations: AMD, adjusted mean difference; Apgar, appearance, pulse, grimace, activity, and respiration; CPAP, continuous -positive -airway -pressure; CRP, C-reactive protein; DCC, delayed cord clamping; EPP, extrauterine placental perfusion; Hb, hemoglobin; IL, interleukin; NA, not applicable; NIPPV, nasal intermittent positive pressure ventilation; RR, relative risk.
SI conversion factors: To convert bilirubin to micromoles per liter, multiply by 17.104; CRP to milligrams per liter or Hb to grams per liter, multiply by 10.
[a] Per-protocol set.
[b]AMDs (with 95% CIs) were estimated by univariate analysis of variance, with cofactors (gestational age: 24 weeks 0 days to 27 weeks 6 days and >27 weeks 6 days and type of pregnancy: singleton and multiple).

eTable 5: Heart Rate and Pulse Oxygen Saturation During Neonatal Transition[a]

		EPP group	DCC group	*AMD (95% CI)[b]*
Heart rate (bpm) 1 min. after birth, mean ± SD, (n)		108.9 ± 37.2, (9)	N/A	N/A
Heart rate (bpm) 2 min. after birth, mean ± SD, (n)		125.3 ± 34.5, (18)	102 ± 38.0, (11)	24.7 (-4.1 to 53.4)
Heart rate (bpm) 3 min. after birth, mean ± SD, (n)		131.4 ± 23.5, (18)	121.8 ± 36.4, (22)	10.5 (-8.7 to 30.0)
Heart rate (bpm) 4 min. after birth, mean ± SD, (n)		125.8 ± 28.3, (18)	131.0 ± 25.8, (24)	-4.8 (-22.2 to 12.7)
Heart rate (bpm) 5 min. after birth, mean ± SD, (n)		135.8 ± 19.1, (17)	133.3 ± 22.1, (25)	2.8 (-10.4 to 16.0)
Heart rate (bpm) 6 min. after birth, mean ± SD, (n)		136.4 ± 24.8, (18)	138.9 ± 20.1, (26)	-1.3 (-15.0 to 12.5)
Heart rate (bpm) 7 min. after birth, mean ± SD, (n)		146.6 ± 16.3, (18)	142 ± 20.8, (26)	5.5 (-6.4 to 17.5)
Heart rate (bpm) 8 min. after birth, mean ± SD, (n)		151.6 ± 15.2, (18)	145.9 ± 18.8, (26)	6.4 (-4.4 to 17.3)
Heart rate (bpm) 9 min. after birth, mean ± SD, (n)		151.7 ± 21, (18)	150.4 ± 18.3, (26)	2.3 (-9.6 to 14.3)
Heart rate (bpm) 10 min. after mean ± SD, (n)		157.1 ± 15.7, (19)	153.9 ± 16.2, (25)	3.6 (-6.4 to 13.6)
SpO_2 (%) 1 min. after birth, mean ± SD, (n)		62.2 ± 22.2, (9)	N/A	N/A
SpO_2 (%) 2 min. after birth, mean ± SD, (n)		56.3 ± 23.4, (17)	138.9 ± 20.1, (11)	5.9 (-10.9 to 22.7)
SpO_2 (%) 3 min. after birth, mean ± SD, (n)		59.6 ± 26.1, (18)	45.7 ± 20.3, (22)	15.1 (0.6 to 29.5)
SpO_2 (%) 4 min. after birth, mean ± SD, (n)		62.2 ± 27.3, (18)	52.1 ± 21.2, (24)	10.7 (-4.7 to 26.2)
SpO_2 (%) 5 min. after birth, mean ± SD, (n)		70.2 ± 26.9, (18)	54.8 ± 17.6, (25)	16.2 (2.4 to 30.0)
SpO_2 (%) 6 min. after birth, mean ± SD, (n)		78.9 ± 27.2, (18)	67.0 ± 19.3, (25)	14.2 (0.7 to 27.8)
SpO_2 (%) 7 min. after birth, mean ± SD, (n)		83.9 ± 18.3, (18)	73.8 ± 20.5, (25)	11.7 (-0.1 to 23.5)
SpO_2 (%) 8 min. after birth, mean ± SD, (n)		89.3 ± 10.6, (18)	77.6 ± 17.8, (25)	12.5 (3.0 to 22.1)
SpO_2 (%) 9 min. after birth, mean ± SD, (n)		91.2 ± 12.2, (18)	81.4 ± 15.2, (26)	10.0 (1.1 to 18.9)
SpO_2 (%) 10 min. after birth, mean ± SD, (n)		91.4 ± 7.8, (19)	85.6 ± 13.1, (25)	6.1 (-0.8 to 13.1)

Abbreviations: AMD, adjusted mean difference; bpm, beats per minute; CI, confidence interval; DCC, delayed cord clamping; EPP, extrauterine placental perfusion; NA, not applicable; SD, standard deviation; SpO2, pulse oxygen saturation.

[a] Per-protocol set.

[b]AMDs (with 95% CIs) were estimated by univariate analysis of variance, with cofactors (gestational age: 24 weeks 0 days to 27 weeks 6 days and >27 weeks 6 days and type of pregnancy: singleton and multiple).

© 2023 Kuehne B et al. *JAMA Network Open.*

eTable 6: Cerebral Regional Oxygenation During Neonatal Transition[a]

		EPP group	DCC group	*AMD (95% CI)[b]*
rcSO2 (%) 1 min. after birth, mean ± SD, (n)		54.5 ± 13.8, (10)	37 ± 0, (1)	0.1 (-53.2 to 53.5)
rcSO2 (%) 2 min. after birth, mean ± SD, (n)		52.9 ± 12.9, (15)	48.6 ± 20.3, (16)	2.5 (-10.3 to 15.2)
rcSO2 (%) 3 min. after birth, mean ± SD, (n)		55.0 ± 12.9, (17)	47.9 ± 18.1, (21)	5.4 (-5.1 to 15.9)
rcSO2 (%) 4 min. after birth, mean ± SD, (n)		62.9 ± 12.9, (17)	48.2 ± 13.8, (20)	12.5 (3.0 to 22.0)
rcSO2 (%) 5 min. after birth, mean ± SD, (n)		66.4 ± 13.4, (17)	49.9 ± 15.8, (19)	14.3 (3.8 to 24.9)
rcSO2 (%) 6 min. after birth, mean ± SD, (n)		74.2 ± 14.0, (18)	55.0 ± 17.3, (21)	18.9 (8.6 to 29.1)
rcSO2 (%) 7 min. after birth, mean ± SD, (n)		78.3 ± 9.2, (18)	61.8 ± 15.6, (23)	16.2 (7.9 to 24.6)
rcSO2 (%) 8 min. after birth, mean ± SD, (n)		75.8 ± 14.7, (19)	64.2 ± 16.1, (23)	11.4 (1.7 to 21.1)
rcSO2 (%) 9 min. after birth, mean ± SD, (n)		81.1 ± 11.6, (19)	70.6 ± 14.5, (23)	10.6 (2.5 to 18.8)
rcSO2 (%) 10 min. after birth, mean ± SD, (n)		81.0 ± 10.8, (19)	74.0 ± 15.2, (22)	7.1 (-1.2 to 15.4)

Abbreviations: AMD, adjusted mean difference; CI, confidence interval; DCC, delayed cord clamping; EPP, extrauterine placental perfusion; rcSO2, regional cerebral oxygen saturation; NA, not applicable; SD, standard deviation.

[a] Per-protocol set.

[b]AMDs (with 95% CIs) were estimated by univariate analysis of variance, with cofactors (gestational age: 24 weeks 0 days to 27 weeks 6 days and >27 weeks 6 days and type of pregnancy: singleton and multiple).

© 2023 Kuehne B et al. *JAMA Network Open.*

eTable 7: Airway Pressure and Fraction Of Inspired Oxygen Levels During Neonatal Transition[a]

		EPP group	DCC group	*AMD (95% CI)*[b]
Airway pressure (cmH2O) 1 min. after birth, mean ± SD, (n)		13.2 ± 0, (18)	12.0 ± 4.2, (2)	1.8 (-4.8 to 8.3)
Airway pressure (cmH2O) 2 min. after birth, mean ± SD, (n)		21.1 ± 4.1, (18)	12.6 ± 4.0, (18)	8.8 (6.3 to 11.3)
Airway pressure (cmH2O) 3 min. after birth, mean ± SD, (n)		23.4 ± 4.3, (19)	17.8 ± 6.0, (18)	5.8 (2.4 to 9.3)
Airway pressure (cmH2O) 4 min. after birth, mean ± SD, (n)		24.3 ± 4.5, (19)	20.7 ± 6.3, (18)	3.8 (0.2 to 7.5)
Airway pressure (cmH2O) 5 min. after birth, mean ± SD, (n)		24.6 ± 4.0, (19)	22.5 ± 4.1, (18)	2.2 (-0.6 to 5.0)
Airway pressure (cmH2O) 6 min. after birth, mean ± SD, (n)		24.7 ± 4.4, (19)	22.6 ± 3.9, (18)	2.2 (-0.7 to 5.0)
Airway pressure (cmH2O) 7 min. after birth, mean ± SD, (n)		25.0 ± 4.4, (19)	22.8 ± 3.7, (18)	2.1 (-0.6 to 4.8)
Airway pressure (cmH2O) 8 min. after birth, mean ± SD, (n)		25.1 ± 4.3, (19)	23.3 ± 3.6, (18)	1.8 (-0.9 to 4.5)
Airway pressure (cmH2O) 9 min. after birth, mean ± SD, (n)		24.8 ± 5.1, (18)	23.3 ± 3.5, (18)	1.4 (-1.7 to 4.4)
Airway pressure (cmH2O) 10 min. after birth, mean ± SD, (n)		24.7 ± 4.8, (19)	23.8 ± 3.3, (17)	0.9 (-2.0 to 3.7)
FiO_2 (%) 1 min. after birth, mean ± SD, (n)		27.4 ± 4.8, (13)	21.0 ± 0, (22)	6.3 (4.1 to 8.4)
FiO_2 (%) 2 min. after birth, mean ± SD, (n)		28.4 ± 5.3, (13)	30.8 ± 8.5, (20)	-2.7 (-8.1 to 2.7)
FiO_2 (%) 3 min. after birth, mean ± SD, (n)		29.7 ± 6.5, (13)	34.4 ± 9.6, (20)	-4.8 (-11.1 to 1.5)
FiO_2 (%) 4 min. after birth, mean ± SD, (n)		34.3 ± 8.7, (13)	37.2 ± 9.8, (20)	-2.8 (-9.7 to 4.2)
FiO_2 (%) 5 min. after birth, mean ± SD, (n)		37.9 ± 10.7, (13)	42.7 ± 16.0, (20)	-5.2 (-15.9 to 5.5)
FiO_2 (%) 6 min. after birth, mean ± SD, (n)		37.5 ± 9.2, (13)	44.2 ± 19.8, (20)	-7.4 (-19.3 to 4.5)
FiO_2 (%) 7 min. after birth, mean ± SD, (n)		35.6 ± 7.4, (13)	43.1 ± 21.4, (20)	-8.5 (-20.8 to 3.9)
FiO_2 (%) 8 min. after birth, mean ± SD, (n)		34.4 ± 8.2, (13)	42.4 ± 21.8, (20)	-8.8 (-21.7 to 4.1)
FiO_2 (%) 9 min. after birth, mean ± SD, (n)		32.4 ± 8.9, (12)	43.6 ± 23.0, (20)	-12.6 (-26.6 to 1.4)
FiO_2 (%) 10 min. after birth, mean ± SD, (n)		31.4 ± 10.5, (13)	41.2 ± 23.4, (19)	-11.0 (-25.1 to 3.0)

Abbreviations: AMD, adjusted mean difference; CI, confidence interval; DCC, delayed cord clamping; EPP, extrauterine placental perfusion; FiO2, fraction of inspired oxygen; NA, not applicable; SD, standard deviation.

[a] Per-protocol set.

[b]AMDs (with 95% CIs) were estimated by univariate analysis of variance, with cofactors (gestational age: 24 weeks 0 days to 27 weeks 6 days and >27 weeks 6 days and type of pregnancy: singleton and multiple).

© 2023 Kuehne B et al. *JAMA Network Open.*
