# Supplementary material for: Extrauterine Placental Perfusion and Oxygenation in Infants With Very Low Birth Weight: A Randomized Clinical Trial
Source: JAMA Netw Open. 2023 Nov 3;6(11):e2340597. doi: 10.1001/jamanetworkopen.2023.40597 (PMC10625045; doi:10.1001/jamanetworkopen.2023.40597)
Supplement: Supplement 3. — Data Sharing Statement [file jamanetwopen-e2340597-s003.pdf]

Data Sharing Statement

Kuehne. Extrauterine Placental Perfusion and Oxygenation in Infants With Very Low Birth Weight: A Randomized Clinical Trial. *JAMA Netw Open*. Published online September 3, 2023. doi:10.1001/jamanetworkopen.2023.40597

Data
Data available: No

Additional Information
Explanation for why data not available: The raw data are available on a reasonable request.
